# Aberrant trafficking of NSCLC-associated EGFR mutants through the endocytic recycling pathway promotes interaction with Src^@^

**DOI:** 10.1186/1471-2121-10-84

**Published:** 2009-11-30

**Authors:** Byung Min Chung, Srikumar M Raja, Robert J Clubb, Chun Tu, Manju George, Vimla Band, Hamid Band

**Affiliations:** 1 Eppley Institute for Cancer and Allied Diseases, University of Nebraska Medical Center, 985950 Nebraska Medical Center, Omaha, NE 68198-5950, USA; 2 Department of Biochemistry & Molecular Biology, University of Nebraska Medical Center, 985870 Nebraska Medical Center, Omaha, NE 68198-5870, USA; 3 Department of Genetics, Cell Biology & Anatomy, College of Medicine, University of Nebraska Medical Center, 985805 Nebraska Medical Center, Omaha, NE 68198-5805, USA; 4 UNMC-Eppley Cancer Center, University of Nebraska Medical Center, 985950 Nebraska Medical Center, Omaha, NE 68198-5950, USA

## Abstract

**Background:**

Epidermal growth factor receptor (EGFR) controls a wide range of cellular processes, and altered EGFR signaling contributes to human cancer. EGFR kinase domain mutants found in non-small cell lung cancer (NSCLC) are constitutively active, a trait critical for cell transformation through activation of downstream pathways. Endocytic trafficking of EGFR is a major regulatory mechanism as ligand-induced lysosomal degradation results in termination of signaling. While numerous studies have examined mutant EGFR signaling, the endocytic traffic of mutant EGFR within the NSCLC milieu remains less clear.

**Results:**

This study shows that mutant EGFRs in NSCLC cell lines are constitutively endocytosed as shown by their colocalization with the early/recycling endosomal marker transferrin and the late endosomal/lysosomal marker LAMP1. Notably, mutant EGFRs, but not the wild-type EGFR, show a perinuclear accumulation and colocalization with recycling endosomal markers such as Rab11 and EHD1 upon treatment of cells with endocytic recycling inhibitor monensin, suggesting that mutant EGFRs preferentially traffic through the endocytic recycling compartments. Importantly, monensin treatment enhanced the mutant EGFR association and colocalization with Src, indicating that aberrant transit through the endocytic recycling compartment promotes mutant EGFR-Src association.

**Conclusion:**

The findings presented in this study show that mutant EGFRs undergo aberrant traffic into the endocytic recycling compartment which allows mutant EGFRs to engage in a preferential interaction with Src, a critical partner for EGFR-mediated oncogenesis.

## Background

Epidermal growth factor receptor (EGFR) is a prototype of receptor tyrosine kinases (RTKs) which control critical cellular responses to extra-cellular growth factors during development and tissue homeostasis [1,2]. Importantly, overexpression of EGFR and/or its ligands is frequently observed in human cancers, and recent studies have identified activating mutations in EGFR as direct determinants of oncogenic transformation in human cancers [3]. For example, missense mutations or small in-frame deletions within the kinase domain, which render EGFR constitutively active, are observed in a subset of patients with non-small cell lung cancer (NSCLC) [4-6]. As mutational activation of EGFR imparts a higher sensitivity to inhibition by EGFR-selective tyrosine kinase inhibitors (TKIs), there is considerable interest in understanding biological mechanisms whereby mutant EGFRs mediate aberrant oncogenic signaling in cancer cells.

While the normal EGFR signaling cascade is initiated by ligand-dependent dimerization and subsequent trans-phosphorylation of tyrosine residues within the cytoplasmic tail of the receptor, constitutively active mutant EGFRs associated with human cancer are thought to engage downstream signaling pathways in a constitutive fashion. Indeed, biochemical analyses have demonstrated that NSCLC-associated EGFR mutants activate signaling through the Erk, Akt, Src and STAT pathways [4,7,8]. A notable finding from these studies has been that certain signaling pathways may be preferentially altered by mutationally-activated EGFRs. For example, phosphoinositide 3-kinase pathway activation by mutant EGFR was found to be highly sensitive to gefitinib, an EGFR tyrosine kinase inhibitor [4,8]. Other studies have indicated a relatively selective activation of Src downstream of mutant EGFRs [7-9].

In the context of Src, use of Src inhibitors [9,10] and mutation of Src-dependent phosphorylation sites within EGFR (Y845) [7,11] have demonstrated a critical role for Src activity in linking mutant EGFRs to activation of several signaling pathways, to cell survival and to mutant EGFR-mediated oncogenic transformation. However, the reasons why certain signaling pathways, such as Src activity-dependent events, might be particularly activated by oncogenic EGFR mutants have not been addressed.

A crucial determinant of events downstream of RTKs such as EGFR is their endocytic traffic [12]. Ligand-dependent internalization of EGFR with subsequent sorting of the internalized receptors for lysosomal degradation has emerged as a major mechanism for termination of signaling. While EGFR endocytosis is a pre-requisite for lysosomal targeting, the latter is not an invariant fate. It has become clear that endocytosed receptors undergo a sorting process whereby internalized receptors can either proceed to the lysosome through a series of vesicular fusion/maturation events or can be recycled back to the plasma membrane [13].

Recent studies have demonstrated that activation-dependent recruitment of the Cbl family of ubiquitin ligases is a major determinant of lysosomal targeting of EGFR [14,15]. Cbl-dependent mono-ubiquitinylation of the cytoplasmic tail of EGFR serves as a signal for receptor sorting to the inner vesicles of the multi-vesicular bodies, a key step in lysosomal targeting of RTKs [16]. Indeed, perturbation of Cbl protein expression or function alters the lysosomal degradation of EGFR and impacts the magnitude and/or duration of downstream signals [15,17]. Additional mechanisms that function either in concert with Cbl-dependent ubiquitin modification, such as sprouty2, Sts-1/Sts-2 and cortactin [18-20], or independently (e.g. Sorting nexins) [21] further contribute to EGFR downregulation through lysosomal targeting.

In contrast to EGF-induced lysosomal targeting of EGFR, TGFα binding appears to promote the recycling of EGFR rather than its lysosomal degradation, correlating with a more potent signaling response [22-24]. Notably, TGFα stimulation is associated with a more transient EGFR-Cbl association and EGFR ubiquitinylation [22]. EGFR heterodimerization with ErbB2, as is often observed in tumor cells, has also been shown to impair lysosomal degradation of EGFR apparently due to increased recycling and/or reduced internalization [25-27].

Given the importance of endocytic trafficking in dictating the lifespan of active EGFR and possibly the quality of downstream signaling events, it is of considerable interest to explore how oncogenic EGFRs traffic. In addition, the ability of mutant EGFRs to hyper-activate certain signaling pathways may be related to altered endocytic trafficking. Consistent with such a possibility, NSCLC-associated EGFR mutants appear to be impaired in their interaction with Cbl [28,29]. More recent studies suggest that specific endocytic routes may dictate the type of biological responses to EGFR stimulation. For example, clathrin-dependent endocytosis appears to be critical for proliferative responses to EGF, whereas clathrin-independent endocytosis appears to primarily promote EGFR degradation [30]. Furthermore, NSCLC-associated EGFR mutants have been shown to undergo EGF-independent internalization when expressed in a murine pro-B cell line [31]. Intracellularly distributed EGFR was also observed in NSCLC cell lines [32]. Here, we examined the subcellular localization of wild-type (wt) EGFR and oncogenic EGFR mutants in normal bronchial epithelial cells and NSCLC cell lines. Findings reported here demonstrate that mutant EGFRs undergo enhanced endocytic recycling and suggests a role of altered endocytic trafficking in mutant EGFR interaction with Src.

## Results

### NSCLC-associated oncogenic EGFR mutants are constitutively endocytosed

**Figure 1:**
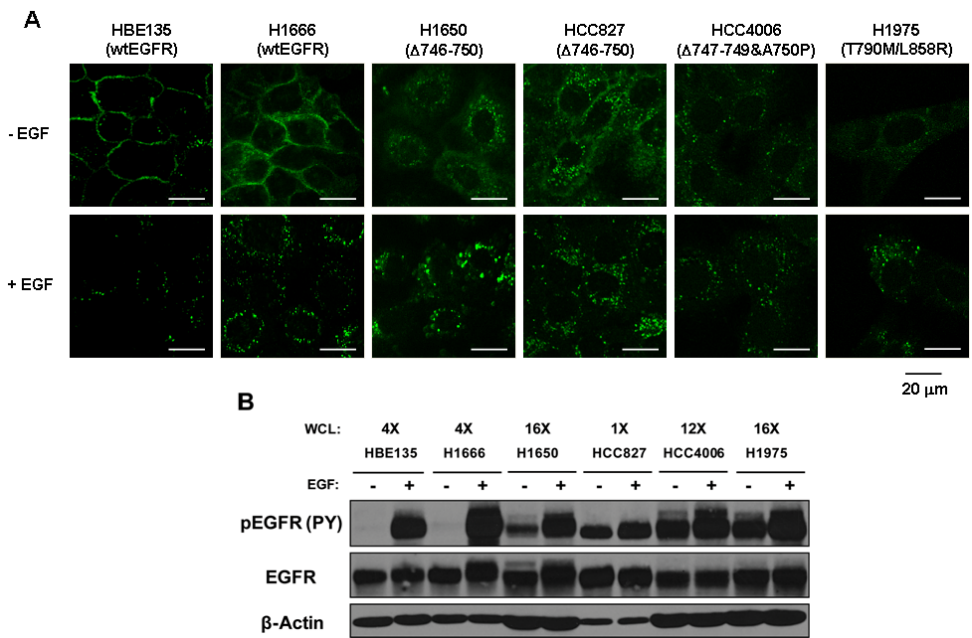
**Constitutive intracellular vesicular localization and phosphorylation of mutant EGF receptors in NSCLC cell lines**. (A) Cells were growth factor-deprived by culturing in D3 medium (HBE135) or 0.1% FBS-containing growth medium (all other cell lines) for 48 hr and then either left unstimulated (- EGF) or stimulated (+ EGF) with 100 ng/ml of EGF for 30 min. Cells were fixed, permeabilized, and immunostained with anti-EGFR antibody 528. Images were acquired under a confocal microscope at the medial plane. Bar represents 20 μm. (B) Cells were growth factor-deprived as in (A) for 48 hr and either left unstimulated (-) or stimulated (+) with 10 ng/ml EGF for 10 min. The indicated amounts of whole cell lysate protein were used for immunoblotting with antibodies against the indicated proteins.

To examine the cell surface versus intracellular (endocytic vesicular) localization of EGFR, we carried out confocal immunofluorescence imaging analyses of a normal bronchial epithelial cell line HBE135 expressing wtEGFR and NSCLC cell lines expressing wtEGFR (H1666) or mutant EGFRs (H1650 and HCC827 expressing EGFR Δ746-750, HCC4006 expressing EGFR Δ747-749/A750P, and H1975 expressing EGFR L858R/T790 M) [4,33]. As anticipated for unstimulated wtEGFR [34], EGFR was essentially exclusively localized at the cell periphery in growth factor-deprived, unstimulated HBE135 and H1666 cell lines, and EGFR was only observed in punctate intracellular vesicles after EGF stimulation of these cells (Figure 1A). In contrast, all of the cell lines expressing mutant EGFRs showed predominantly punctate EGFR staining with a much lower level of peripheral cell surface staining when examined without EGF stimulation; the intracellular punctate staining increased further upon EGF stimulation (Figure 1A). Mutant EGFRs ectopically expressed in HBEC cell lines as EGFR-GFP chimeras showed similar results, indicating that the constitutive intracellular localization of mutant EGF receptors observed in Figure 1 was not due to cell type specificity (Additional File 1A). Correlating with the immunofluorescence results, mutant EGFRs displayed high levels of basal phosphorylation (with further increase upon EGF stimulation) whereas the phosphorylation of wtEGFR was observed only upon EGF stimulation (Figure 1B and Additional File 1B), as expected from previous biochemical analyses [4,35].

**Figure 2:**
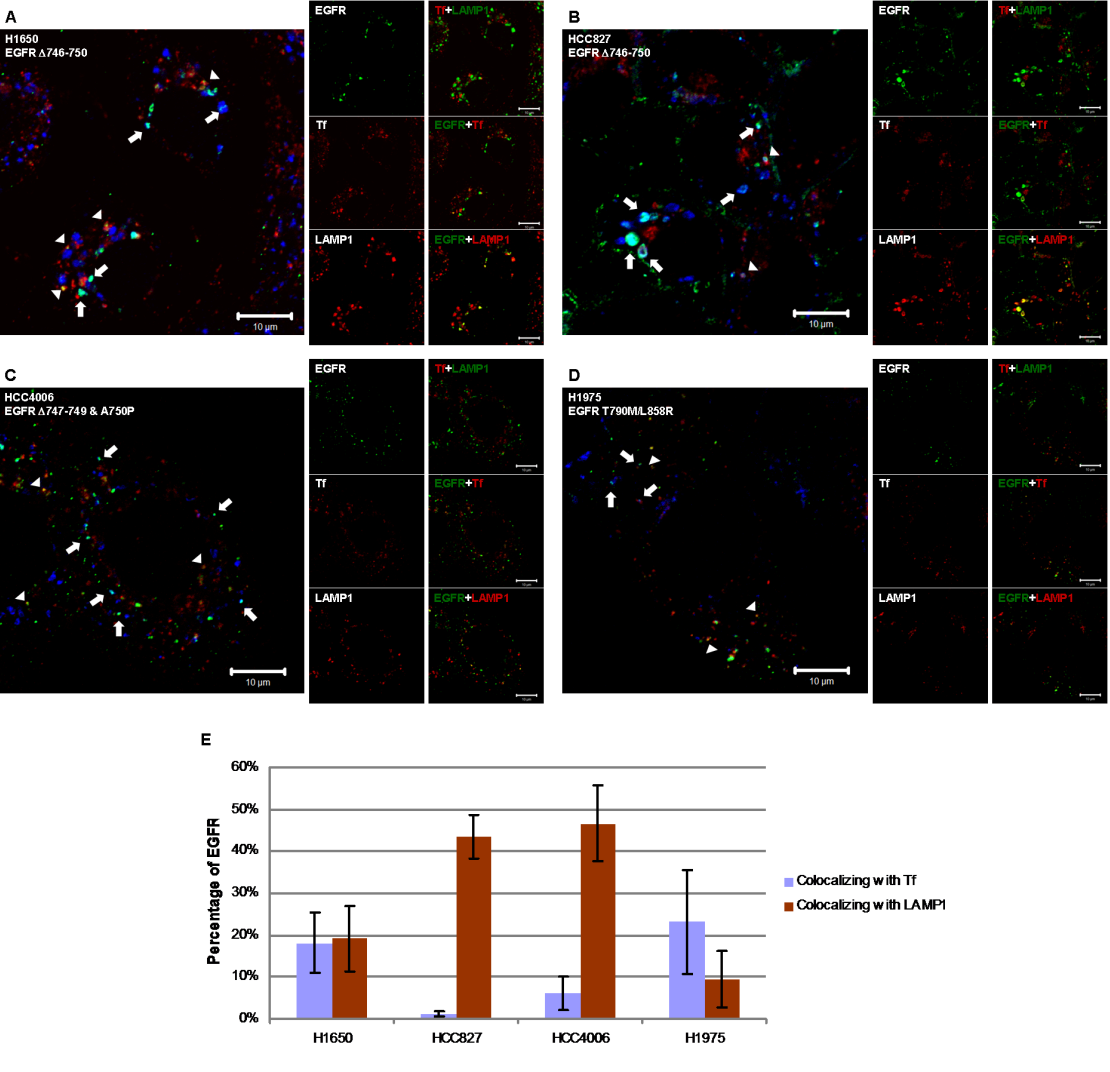
**Constitutively endocytosed mutant EGF receptors colocalize with early/recycling endosomal marker transferrin and late endosomal/lysosomal marker LAMP1**. NSCLC cell lines H1650 (A), HCC827 (B), HCC4006 (C) or H1975 (D) were serum-starved for 48 hr and incubated with 10 μg/ml of AF546-transferrin (Tf) (red) for 30 min at 37°C. Cells were fixed, permeabilized and immunostained with anti-EGFR antibody 528 (green), followed by anti-LAMP1 antibody H4A3 (blue). Images were acquired under a confocal microscope at the medial plane. Arrows indicate EGFR and LAMP1 colocalization, and arrowheads indicate EGFR and labeled-transferrin colocalization. Bars represent 10 μm. (E) Colocalization coefficients for the green channel (EGFR) from (A), (B), (C), and (D) were obtained using LSM510 Image Examiner software, and depicted as percentages of EGFR colocalizing with Tf or LAMP1.

To determine the identity of intracellular vesicles in which the mutant EGFRs reside, we loaded cells with fluorescently-labeled transferrin, an early and recycling endosomal marker, followed by immunostaining for EGFR and LAMP1, the latter as a late endosomal/lysosomal marker (Figure 2A-D). Labeled transferrin loading for 30 min allowed for an examination of both early and recycling endosomes [13]. Mutant EGFRs in all of the cell lines examined (H1650, HCC827, HCC4006 and H1975) colocalized both with transferrin and LAMP1, albeit at different levels in the steady-state conditions (Figure 2E). This data indicates that under steady-state conditions the mutant EGFRs are located in multiple endosomal compartments within the cell.

Incubation of cells at 16°C allows for continued internalization from the cell surface but blocks further progression of endocytosed receptors and cargo along the endocytic pathway and into the endocytic recycling compartments, resulting in enhanced accumulation in sorting endosomes [36]. Indeed, when NSCLC cell lines were incubated at 16°C, mutant EGFRs showed enhanced colocalization with labeled transferrin (Additional File 2). This result further suggests that mutant EGFRs transit along a transferrin-positive sorting compartment.

### Treatment with monensin results in the accumulation of mutant EGFR, but not wtEGFR, in a perinuclear endocytic compartment

While our colocalization analyses demonstrated that constitutively endocytosed mutant EGFRs do transit to the lysosome, recent reports indicate that mutant EGFRs show reduced ligand-induced ubiquitinylation and degradation [28,37] which could promote their entry into the endocytic recycling pathway; colocalization of mutant EGFRs with transferrin (Figure 2) is consistent with this idea. To further test this possibility, we examined the localization of mutant EGFRs after treating cells with monensin, an agent that has been shown to inhibit exit of internalized receptors and other endocytic cargo from sorting endosomes and the endocytic recycling compartment [34,38,39]. To demonstrate the ability of monensin to inhibit the cargo exit from the endocytic recycling compartment, we first assessed its effects on transferrin recycling in the NSCLC cell line H1666. As expected, labeled transferrin exit out of the perinuclear endocytic recycling compartment was essentially complete within the 60 min chase period; however, monensin treatment markedly delayed this process (Additional File 3A).

**Figure 3:**
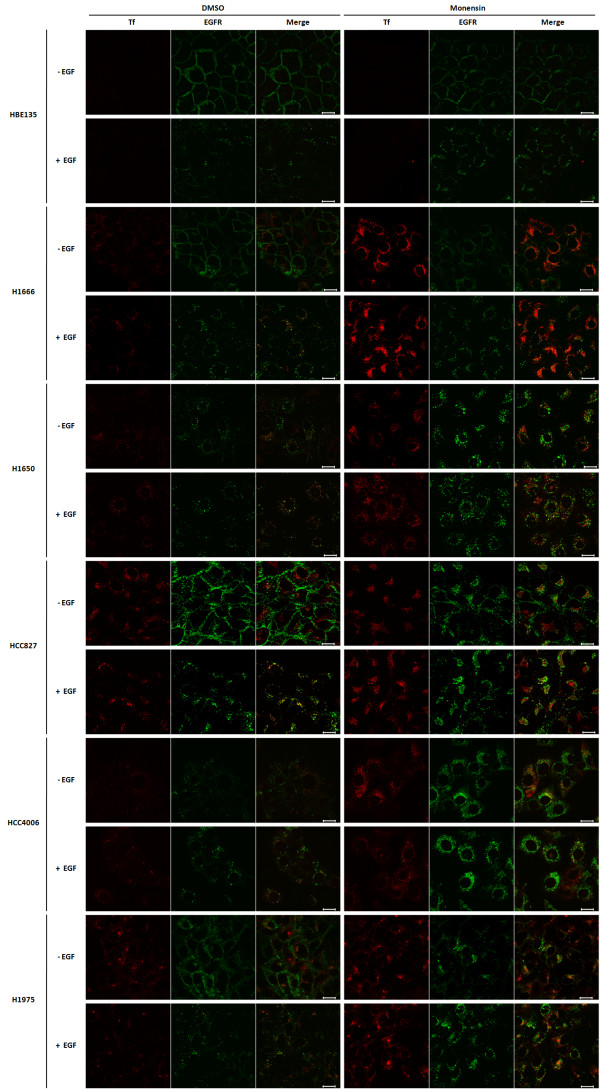
**Monensin-treatment increases the accumulation of mutant EGF receptors in perinuclear endocytic vesicles**. Cells were growth factor-deprived in D3 medium (HBE135) or 0.1% FBS-containing growth medium (all other cell lines) for 48 hr and then preincubated with DMSO or 10 μM monensin for 3 hr. Cells were loaded with 10 μg/ml AF546-Tf (Tf) (red) for 45 min at 37°C (with continuation of DMSO or monensin treatment). This was followed by incubation with (+ EGF) or without (- EGF) 10 ng/ml EGF for 30 min with continued DMSO or monensin treatment. Cells were fixed, permeabilized, and immunostained with anti-EGFR antibody 528 (green). Images were acquired under a confocal microscope at the medial plane. Bars represent 20 μm.

To assess the impact of recycling inhibition on mutant versus wild-type EGFR, we carried out concurrent EGF stimulation and labeled transferrin chase in HBE135 and NSCLC cell lines with or without pre-incubation in monensin (Figure 3). While the relatively low uptake of transferrin in the HBE135 cell line did not permit a clear assessment of transferrin accumulation upon monensin treatment, all of the NSCLC cell lines, including the wtEGFR-expressing cell line H1666, showed a marked increase in perinuclear labeled transferrin staining in the presence of monensin, indicating an effective inhibition of cargo exit from the endocytic recycling compartment. Importantly, monensin treatment induced the perinuclear accumulation of EGFR in H1650, HCC827, HCC4006 and H1975 cell lines bearing mutant EGFRs, but not detectably in HBE135 and H1666 cell lines bearing the wtEGFR, either in the presence or absence of EGF stimulation. Similar perinuclear mutant EGFR accumulation was observed upon monensin treatment of cells grown in regular growth media without any growth factor deprivation or EGF stimulation (Additional File 3B), and also in HBEC cell lines stably expressing ectopic mutant EGF receptors (Additional File 3C).

NSCLC-associated mutant EGFRs have been shown to attain sensitivity to Hsp90 inhibitor 17-(allylamino)-17-demethoxygeldanamycin (17-AAG) which targets the related receptor ErbB2 to degradation by enhancing its lysosomal targeting [37,40,41]. Notably, the presence of monensin prevented the lysosomal targeting of mutant EGFR and its degradation induced by 17-AAG (Additional File 4). 17-AAG treatment resulted in a decrease in mutant EGFR staining, indicating that mutant EGFR was targeted for degradation in the lysosomes. The 17-AAG-induced mutant EGFR downregulation was inhibited in monensin-treated cells and intracellular punctate staining of EGFR could still be observed. This is consistent with the concurrent effect of monensin to block traffic towards the lysosome [42].

To rule out the possibility that the perinuclear accumulation of mutant EGFRs may reflect an overall increase in the level of EGFR, we compared the EGFR expression levels in cells treated with DMSO versus monensin (Additional File 5A). Neither the overall EGFR levels nor the overall level of EGFR phosphorylation, as determined using anti-phosphotyrosine (PY) and anti-phospho-EGFR antibodies specific to pY845 and pY1173, showed a gross change upon monensin treatment (Additional File 5A).

### Mutant EGFR colocalizes with markers of endocytic recycling compartment

**Figure 4:**
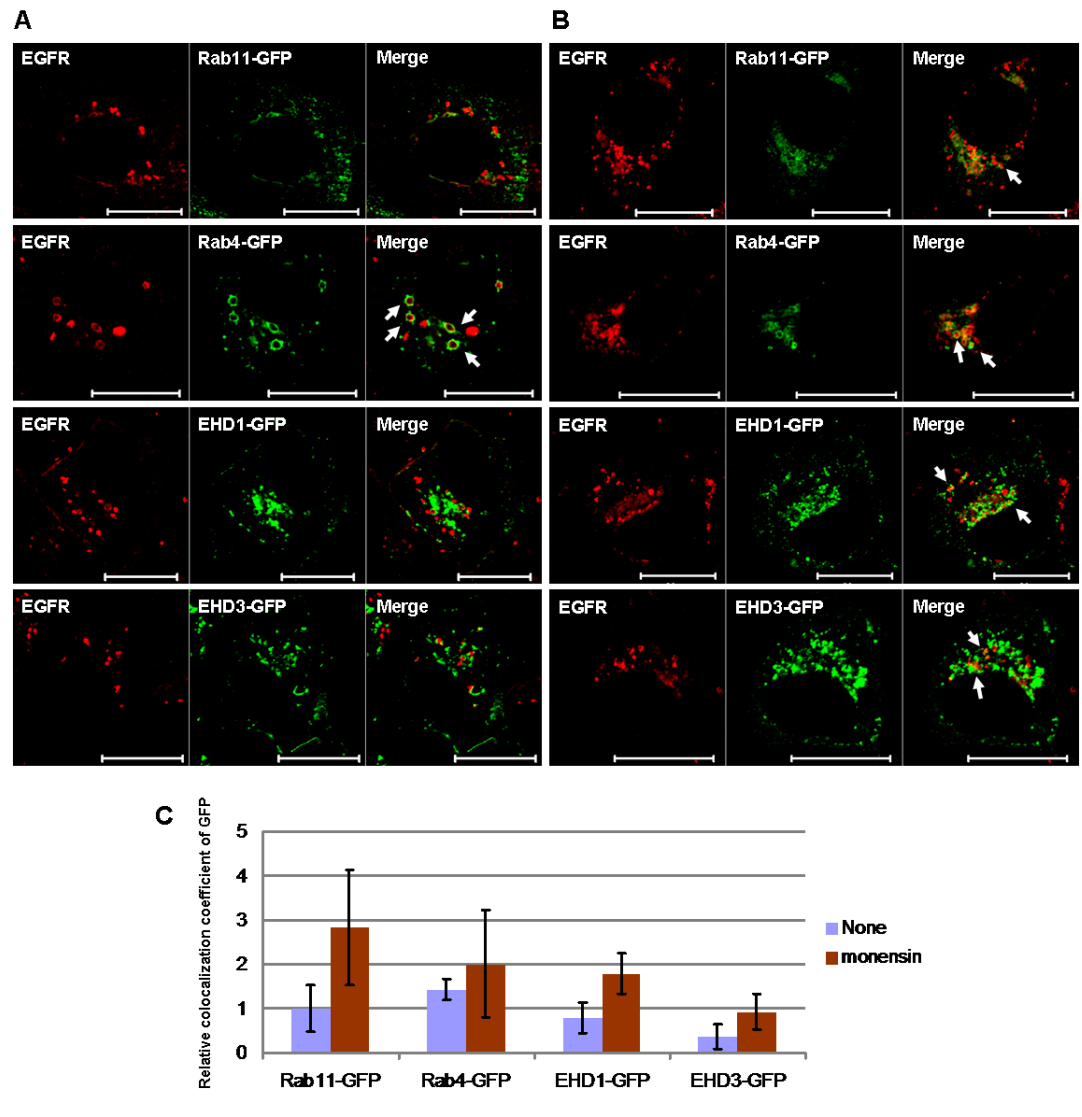
**Mutant EGF receptor colocalization with endocytic recycling compartment markers**. HCC827 NSCLC cell line was transiently transfected with Rab11-GFP, Rab4-GFP, EHD1-GFP or EHD3-GFP constructs. After 48 hr, cells were either left untreated (A) or treated with 10 μM monensin for 2 hr (B). Cells were fixed, permeabilized, and immunostained with anti-EGFR antibody (528) (red). Images were acquired under a confocal microscope at the medial plane. Arrows indicate mutant EGFR colocalization with GFP-tagged proteins (green). Bars represent 20 μm. (C) Colocalization coefficients for the green channel (GFP) were obtained using LSM510 Image Examiner software and normalized to the colocalization coefficient from Rab11-GFP without any treatment (none).

Enhanced colocalization of mutant EGFRs with transferrin at 16°C together with perinuclear accumulation upon monensin treatment suggested that mutant EGFRs preferentially transit through the endocytic recycling compartment. Therefore, we carried out confocal imaging studies to assess if the constitutively endocytosed mutant EGFRs show colocalization with endocytic recycling compartment markers. Rab proteins are known to regulate various steps in endocytic traffic: Rab4 regulates fast/direct recycling from the early endosomes to the plasma membrane, while Rab11 regulates recycling from the deeper perinuclear recycling compartments [13,36]. The newly identified EHD protein family also controls endocytic recycling, with EHD1 functioning in the endocytic recycling compartment and EHD3 in the early endosomes [43,44]. The mutant EGFR-expressing cell line HCC827 was transiently transfected with expression vectors coding for GFP-tagged Rab11, Rab4, EHD1 or EHD3; after 48 hr, the cells were fixed and immunostained with an anti-EGFR antibody (Figure 4A). Partial colocalization of mutant EGFR with markers of early and recycling endosomes was observed (Figure 4A), and notably, enlarged GFP-positive vesicles were observed surrounding the EGFR-positive punctate structures (arrows), especially in cells transfected with Rab4-GFP. Monensin treatment further increased the appearance of these enlarged vesicles for all of the early and recycling endosomal markers as well as colocalization between these markers and mutant EGFR (Figure 4B and 4C), consistent with the monensin-induced block of exit from the endocytic recycling compartments. The confocal colocalization studies therefore further support the conclusion that mutant EGFRs traffic through the endocytic recycling compartments.

### Src association with mutant EGF receptors in the endocytic recycling compartment

**Figure 5:**
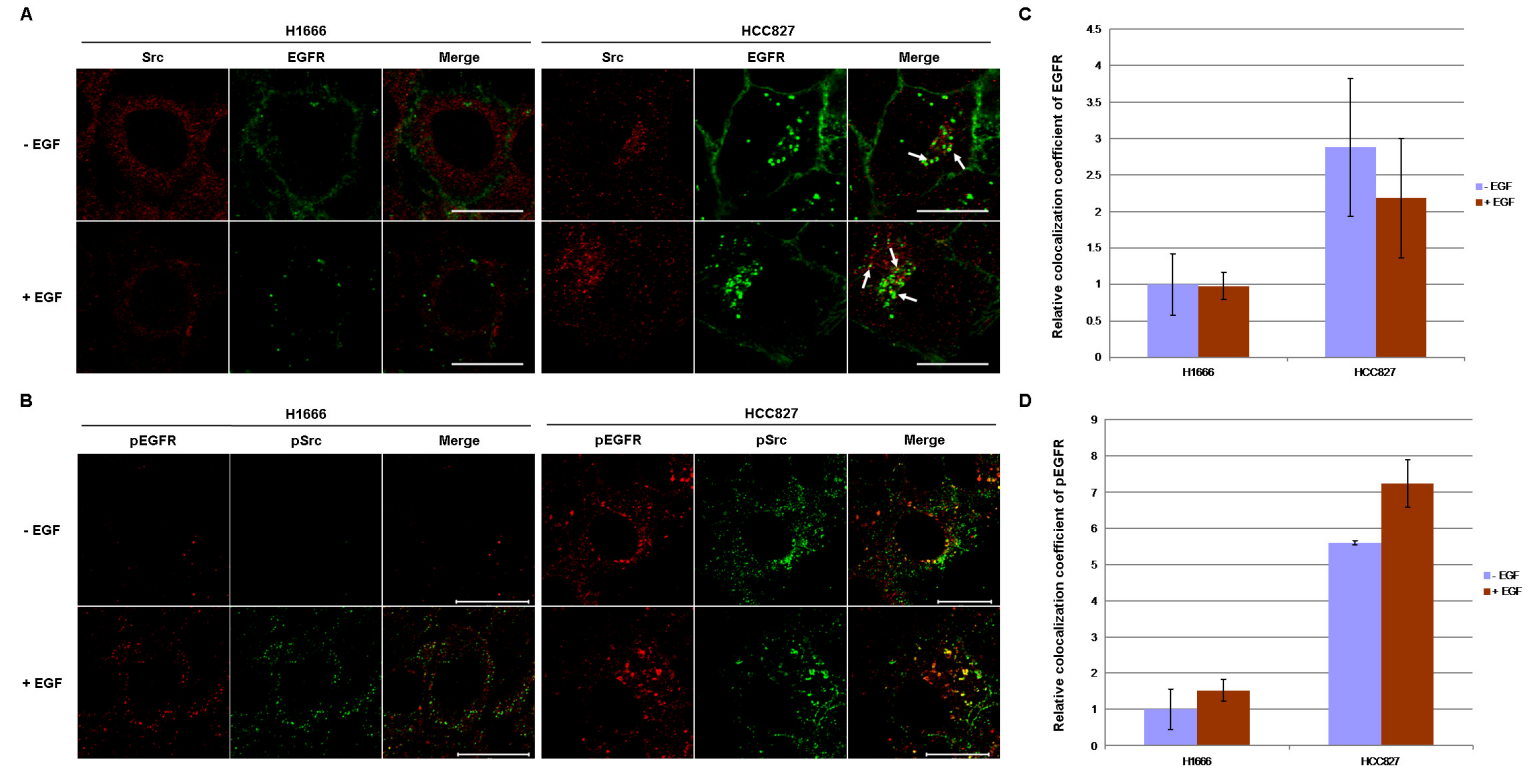
**Mutant EGF receptors colocalize with Src in NSCLCcell lines**. Cells were growth factor-deprived in 0.1% FBS-containing growth medium for 48 hr and then either left unstimulated (- EGF) or stimulated (+ EGF) with 100 ng/ml of EGF for 10 min. Cells were fixed, permeabilized, and immunostained with (A) anti-EGFR antibody 528 (green) followed by anti-Src antibody SRC2 (red) staining or (B) anti-phospho-EGFR antibody (red) followed by anti-phospho-Src antibody (green). Images were acquired under a confocal microscope at the medial plane. Arrows indicate EGFR and Src colocalization. Bars represent 20 μm. (C) Colocalization coefficients for the green channel (EGFR) from (A), and (D) colocalization coefficients for the red channel (pEGFR) from (B) were obtained using LSM510 Image Examiner software and normalized to the colocalization coefficient from unstimulated (- EGF) H1666 cells.

Constitutive localization of mutant EGFRs in the endocytic recycling compartments (Figure 4) could allow preferential interaction of mutant EGFRs with certain signaling pathways. A particular EGFR-relevant signaling partner in this regard is Src, which is known to localize on endocytic vesicles including the endocytic recycling compartment [45,46]. Furthermore, mutant EGFRs show increased constitutive association with Src, and Src-EGFR interaction plays an important role in mutant EGFR-induced oncogenic transformation [7,9,47]. Therefore, we examined the relative subcellular localizations of EGFR and Src in NSCLC cell lines that were serum-starved and then left untreated or treated with EGF for 10 min (Figure 5A). As observed above (Figure 1A), EGF-deprived H1666 cells (wtEGFR) showed predominantly surface-localized EGFR staining, whereas HCC827 cell line showed constitutive localization of mutant EGFR in intracellular vesicles. Anti-Src staining showed a predominantly perinuclear vesicular staining, consistent with previous reports [45,46]. While wtEGFR was internalized upon EGF stimulation of H1666 cells as expected, we observed very little, if any, colocalization between EGFR and Src under these conditions. In contrast, constitutively internalized mutant EGFR in the HCC827 cell line exhibited enhanced colocalization with Src when compared to wtEGFR in the H1666 cell line (arrows, Figure 5A and 5C). Enhanced colocalization between phospho-EGFR and phospho-Src was also observed in the HCC827 cell line, indicating that constitutive active mutant EGFR interacts with activated Src in endosomal compartments (Figure 5B and 5D). Similar results were seen with the H1650, HCC4006 and H1975 cell lines (data not shown).

**Figure 6:**
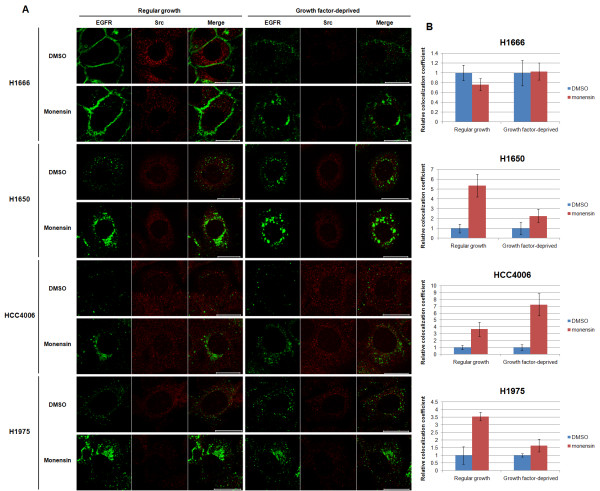
**Monensin treatment enhances mutant EGFR-Src colocalization in endocytic vesicles**. (A) Cells were either growth factor-deprived or left in regular growth medium for 48 hr and incubated with DMSO or 10 μM monensin for 3 hr. Cells were fixed, permeabilized, and immunostained with anti-EGFR antibody 528 (green) followed by anti-Src antibody SRC2 (red) staining. Images were acquired under a confocal microscope at the medial plane. Bars represent 20 μm. (B) Colocalization coefficients for the red channel (anti-Src staining) were obtained using LSM510 Image Examiner software and normalized to the colocalization coefficient from DMSO controls.

As monensin treatment increased the mutant EGFR accumulation in the perinuclear endocytic vesicles (Figure 3), we examined the extent of EGFR and Src colocalization in cells treated with monensin. Treatments were carried out as in Figure 3, and cells were then immunostained for EGFR and Src (Figure 6A). Monensin treatment increased the perinuclear accumulation of mutant but not wild-type EGFR, similar to results in Figure 3. Notably, Src showed an enhanced colocalization with mutant EGFRs that accumulated in perinuclear vesicles; quantification of the relative colocalization (as a colocalization coefficient of Src) confirmed the enhancement upon monensin treatment (Figure 6B). Thus, mutant but not wild-type EGFR displayed enhanced colocalization with Src in endocytic vesicles, and such colocalization was further enhanced by inhibiting the exit of EGFR from the endocytic recycling compartment with monensin.

### Monensin treatment enhances the mutant EGFR-Src association

**Figure 7:**
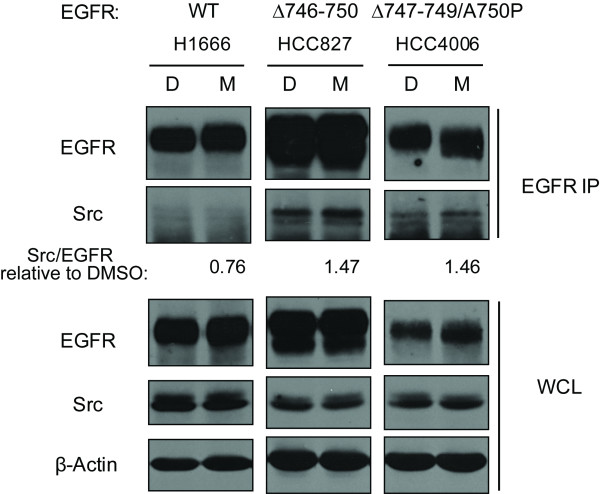
**Monensin treatment enhances the mutant EGFR-Src association**. Cells were growth factor-deprived for 48 hr and incubated with DMSO (D) or 10 μM monensin (M) for 3 hr. 1 mg aliquots of cell lysate proteins were immunoprecipitated with anti-EGFR antibody 528 and resolved together with 50 μg aliquots of whole cell lysates followed by immunoblotting with antibodies against the indicated proteins. The levels of EGFR and Src bands on blots were quantified using densitometry and immunoprecipitated Src relative to immunoprecipitated EGFR levels (Src/EGFR) were determined using the ImageJ software. Src/EGFR ratios in monensin-treated cells versus DMSO-treated cells are noted.

In view of the increased colocalization of mutant EGFR and Src in monensin-treated cells, and recent findings that mutant EGFRs constitutively complex with Src [7,48], we asked if monensin-induced block of EGFR exit from the endocytic recycling compartment influences mutant EGFRs and Src association. Cells processed essentially as for confocal imaging in Figure 6 were used to carry out co-immunoprecipitation analyses to assess EGFR and Src association (Figure 7). In parallel with the increased mutant EGFR and Src colocalization seen in Figure 6, the amounts of Src that co-immunoprecipitated with mutant EGFRs, but not with wtEGFR, were enhanced in the presence of monensin. Similar results were seen when cells were grown and treated with monensin in regular growth media (data not shown).

## Discussion

The outcome of RTK signaling involves a balance between various stimulatory and inhibitory mechanisms which in turn determine both the strength and duration of signals that are transmitted through networks of signaling cascades [49]. In this respect, endosomal sorting plays a key role in the regulation of EGFR signaling [12].

NSCLC-associated kinase domain mutations in EGFR promote its constitutive activation, and a number of studies have focused on delineating the signaling pathways whose activation contributes to oncogenesis [3]. The outcome of EGFR signaling is intimately linked to its endocytic traffic, which is normally triggered by ligand-induced dimerization [50] and phosphorylation-dependent as well as phosphorylation-independent recruitment of endocytic machinery components [51,52]. The nature of endocytic trafficking of NSCLC-associated EGFR mutants and any relationship of altered traffic with oncogenic signaling remain poorly understood. Here, we have used NSCLC cell lines to demonstrate that oncogenic mutant EGFRs, but not wtEGFR, are constitutively endocytosed (Figure 1). Mutant EGFRs were found to localize in early and recycling endosomes based on colocalization with labeled transferrin (Figure 2) and GFP-tagged Rab4, Rab11, EHD1 and EHD3 proteins (Figure 4). Notably, blocking the exit of endocytosed receptors from endocytic recycling compartments with monensin led to a marked accumulation of mutant EGFR in a perinuclear endocytic compartment (Figure 3) and increased its colocalization with markers of sorting and endocytic recycling compartments (Figure 4B). Thus, these findings strongly suggest that mutant EGFRs transit through the endocytic recycling compartment.

Importantly, enhanced EGFR-Src as well as activated EGFR/phospho-Src colocalization was observed in endocytic vesicles of a mutant EGFR-expressing cell line (Figure 5). Furthermore, monensin treatment increased the colocalization of mutant EGFRs with Src in the perinuclear endosomal compartment (Figure 6) and enhanced the biochemical association between mutant EGFRs and Src (Figure 7). Given the emerging evidence for a critical role of the constitutive engagement of Src-mediated signaling pathways in mutant EGFR-dependent oncogenesis [7,9,10], our results suggest a potentially important role of altered endocytic trafficking in the oncogenic behavior of mutant EGFRs.

In view of the critical role of ligand-induced internalization and lysosomal targeting in limiting EGFR signaling, the constitutive activation of downstream signaling pathways by NSCLC-associated mutant EGFRs has generated interest into potential alterations of endocytic trafficking. For example, given the critical role for the Cbl-family of ubiquitin ligases in orchestrating EGFR ubiquitinylation and subsequent lysosomal sorting, it is notable that a recent analysis of NSCLC-associated mutant EGFRs showed reduced Cbl-dependent lysosomal downregulation [28,29,53]. However, another study in an NSCLC cell line reported that mutant EGFR traffics into lysosomes upon EGF stimulation [54]. The present study extends beyond these observations by demonstrating that mutant EGFRs traffic through the endocytic recycling compartment. Our observations, that mutant EGFRs localize to the lysosomes (Figure 2) and block of their endocytic transit by low temperature incubation (Additional File 2) or monensin treatment led to reduced degradation (Additional File 4), are consistent with the idea of mutant EGFRs trafficking into lysosomes. However, our observations do not contradict the defective ubiquitin-dependent trafficking of mutant EGFRs reported by Shtiegman et al., and others [28,29,37], as our studies did not address this issue.

Whether the increased transit through the endocytic recycling compartment is an intrinsic property of mutant EGFRs or is a secondary consequence of their reportedly reduced interaction with Cbl and ubiquitin-mediated lysosomal sorting machinery are important questions that will need to be addressed through appropriate manipulations in NSCLC cells as well as the use of ectopic gene expression approaches. In this regard, it is noteworthy that conditions that prevent EGFR interaction with Cbl or its Cbl-dependent ubiquitinylation lead to a more prolonged stay of EGFR in early/recycling endosomal compartments [15,55,56]. Physiologically, ligands such as TGFα that promote EGFR recycling rather than lysosomal degradation appear to engage the Cbl and ubiquitin machinery more transiently [22,57]. In addition to altered ubiquitinylation of mutant EGFRs, other defects in their signaling or protein-protein interactions could contribute to their propensity to enter the endocytic recycling compartment. For example, deubiquitinylating enzymes [58,59] as well as other factors (e.g. secretory membrane carrier protein SCAMP3) can regulate EGFR recycling versus lysosomal degradation [13,60]. Future studies to elucidate whether or not mutant EGFRs might aberrantly interact with such proteins will therefore be of considerable interest.

NSCLC-associated mutant EGFRs (both gefitinib-sensitive deletion mutants and gefitinib-resistant L858R/T790 M mutant) are constitutively active and constitutively endocytosed (Figure 1 and Additional File 1). Recent studies have demonstrated that NSCLC-associated kinase domain mutations promote constitutive dimerization of EGFR [61]. As dimerization is critical to EGFR endocytosis and may promote internalization in a kinase-dependent [2] or kinase-independent [50,62] manner, constitutive dimerization may play an important role in the transit of mutant EGFRs into the endocytic recycling compartment. In this context, our observations using kinase inhibitors indicate that the kinase activity of EGFR is not essential for the constitutive endocytic localization of mutant EGFR (Additional File 6A). The intracellular localization of mutant EGFR was also unaffected by Src inhibitor PP2, indicating that there may be another determinant of constitutive endosomal localization of mutant EGFRs.

Transit of the constitutively-active mutant EGFR through the endocytic recycling compartment is likely to be biologically relevant. Analyses of EGFR as well as other RTKs have demonstrated that endocytic recycling, in addition to returning the internalized receptors for additional rounds of ligand-binding and signaling, can directly participate in signaling events [12]. For example, inhibition of EGFR internalization reduced the level of activation of Akt and MAPK downstream of the receptor [30,63]. Notably, initiation of EGFR activation directly at the level of endosomes has been shown to be sufficient to activate Erk and Akt, as well as promote cell survival and proliferation [34,64]. However, monensin treatment did not enhance Erk, Akt and STAT3 phosphorylation levels (Additional File 5B). The lack of monensin effect on downstream signaling is likely to reflect its ability to affect multiple endocytic compartments and/or its effects on other cellular processes [38,42,65]. Nevertheless, our observations of EGFR and Src colocalization and association are consistent with a role of signaling at the level of the endocytic recycling compartment in the biology of mutant EGFR.

Our analyses of mutant EGFR recycling in the context of Src were based on prior evidence that Src-dependent signaling is critical for EGFR-mediated oncogenesis; this has been established *in vitro *using Src inhibitors as well as mutational approaches [7], and Src is overexpressed or hyperactive in NSCLC as well as other cancers where EGFR mutations or overexpression have been implicated in oncogenesis [9,10]. Importantly, Src has been shown to localize to endosomes [46], and recent studies have shown that Src specifically localizes on recycling endosomes [45,66]. Thus, it appears plausible that mutant EGFRs, by virtue of their transit through the endocytic recycling compartment, may gain enhanced access to Src, providing a potential explanation for the higher level of constitutive Src-mutant EGFR association [7,48]. Confocal image analyses indeed support this possibility, as Src and mutant EGFRs show a detectable colocalization (versus essentially little detectable colocalization of Src with wtEGFR) (Figure 5); moreover, this colocalization was further increased by inhibiting the exit of EGFR from the endocytic recycling compartment using monensin (Figure 6). Also, a predominant pool of activated EGFR colocalized with activated Src (Figure 5), and Src inhibitor slightly decreased the mutant EGFR-Src association (additional File 6), which suggest that Src activity might be important for colocalization and association with mutant EGFR. In a different study, a Src inhibitor did not inhibit mutant EGFR-Src association [48]. The difference between the two studies may be due to different types of inhibitor and/or cell lines tested.

Rather interestingly, monensin treatment led to a higher level of biochemically detectable EGFR-Src complexes (Figure 7). This, together with higher constitutive Src-mutant EGFR association, suggests the likelihood that Src-mutant EGFR complexes are either formed or more stable in the endocytic recycling compartment. As Src-dependent signaling is critical for mutant EGFR-mediated oncogenic transformation [7], these findings suggest that altered trafficking of mutant EGFRs into the endocytic recycling compartment may contribute to their oncogenic behavior. Further studies to perturb the endocytic recycling of oncogenic EGFR mutants should help address the biological role of the altered endocytic trafficking identified here.

It has been reported that a gefitinib-resistant version of H1650 NSCLC cell line showed increased internalization of EGFR upon ligand stimulation when compared to the parental gefitinib-sensitive cell line [67]. Notably, the wtEGFR in the gefitinib-resistant cell line did not undergo ligand-induced lysosomal sorting, even though the receptor was found in endocytic vesicles [54]. In our analyses, we observed a comparable pattern of subcellular localization and endocytic trafficking of gefitinib-sensitive (deletion) and gefitinib-resistant (L858R/T790 M) EGFR mutants (Figures 1, 2, 3 and Additional Files 2 and 3). Similarly, both gefitinib-resistant H1975 and gefitinib-sensitive H1650 cell lines showed delayed internalization of labeled EGF in comparison to the wtEGFR-expressing cell line H358 [28]. However, there were subtle differences among different cell lines harboring mutant EGFRs in the perinuclear accumulation of the mutant EGFR induced by monensin in the regular growth condition (Additional File 3B); the perinuclear accumulation of EGFR was dramatic in HCC827 and HCC4006, intermediate in H1650, and not readily apparent in H1975. Similarly, quantitative assessments of EGFR localization under steady-state conditions (Figure 2E) suggested differences between different NSCLC lines: the mutant EGFR is evenly divided between Tf-positive and LAMP1-positive vesicles in H1650, HCC827 and HCC4006 showed much more mutant EGFR in LAMP1-positive than in Tf-positive vesicles; and gefitinib-resistant mutant EGFR in H1975 colocalized more with Tf than with LAMP1. In addition, H1650 cell line displayed more sensitivity to EGF than other mutant EGFR-expressing cell lines (Figure 1 and Additional File 5A). Whether EGFR expression levels, the nature of EGFR mutations, and/or activities of EGFR regulatory factors such as Src, Cbl or PTEN, which has been shown to be absent in the H1650 cell line [68], might contribute to the differences in the localization of mutant EGFR and their endocytic trafficking remain open questions. While it is possible that altered endocytic trafficking of EGFR relates to gefitinib resistance, extensive future studies are needed to determine if this is the case.

## Conclusion

In summary, the results presented here show that mutant EGFRs in NSCLC cell lines constitutively transit through the sorting and endocytic recycling compartments. Impairment of EGFR exit from the endocytic recycling compartment enhances the mutant EGFR colocalization with Src in the endocytic recycling compartments and increases the Src-mutant EGFR association. Given the critical role of Src-mediated signaling in mutant EGFR-mediated oncogenic transformation, our findings suggest a potentially important role for altered endocytic trafficking in the biology of NSCLC-associated EGFR mutants.

## Methods

### Constructs

The EHD1-GFP and EHD3-GFP expression constructs in the pcDNA-DEST47 vector were described previously [43]. The Rab4-GFP and Rab11-GFP expression constructs in the EGFPN1 vector [69] were provided by Dr. Victor Hsu (Brigham and Women's Hospital, Harvard Medical School, Boston, MA). The lentiviral expression vectors pLenti6-V5-UbC GFP, wtEGFR-GFP, EGFR L858R-GFP, and EGFR Δ746-750-GFP were generated using the Gateway cloning technology (Invitrogen, Carlsbad, CA). EGFR was PCR amplified from pcDNA 3.1 EGFR using primers CACCATGCGACCCTCCGGGACGG and TGCTCCAATAAATTCACTGCTTTG, and the amplified fragment was inserted into pENTR/SD/D-TOPO vector using the pENTR/SD/D-TOPO cloning kit (Invitrogen). LR recombination reaction was performed to insert EGFR into the pDEST47 vector to generate an EGFR-GFP chimera. EGFR-GFP was PCR amplified using primers CACCATGCGACCCTCCGGGACGG and TTATTTGTAGAGCTCATCCATGCC, inserted into the pENTR vector, and finally into the pLenti6-V5-UbC vector. PLenti6-V5-UbC EGFR L858R-GFP, and EGFR Δ746-750-GFP were generated using the QuikChange II XL Site-Directed Mutagenesis Kit (Strategene, La Jolla, CA) as previously described [7]. All PCR reactions were performed using the QuikChange II XL Site-Directed Mutagenesis Kit following the manufacturer's instructions.

### Human bronchial epithelial cell line immortalization and lentiviral transfection

Primary normal bronchial epithelial cells (HBEC) obtained from a bronchoscopic biopsy sample were provided by Dr. Ravi Salgia (University of Chicago). Cells were transduced with retroviral supernatants of human papilloma virus (HPV) E6 and E7 and selected for several weeks to generate an immortalized human bronchial epithelial cell (HBEC) cell line. Lentiviral supernatants generated as per Gateway cloning technology protocol were used to make the HBEC cell line stably expressing pLenti6-V5-UbC vector, GFP, wtEGFR-GFP, EGFR L858R-GFP, or EGFR Δ746-750-GFP.

### Cell culture and transient transfection

Immortalized bronchial epithelial cell line HBE135 (ATCC, Manassas, VA) and HBEC were grown in the DFCI-1 medium described in Band et al. [70]. NSCLC tumor cell lines H1666, H1650, HCC827, HCC4006 and H1975 (ATCC) were grown in RPMI-1640 medium (Invitrogen, Carlsbad, CA) containing 5% fetal bovine serum (FBS, Hyclone Inc., Logan, UT), 20 mM HEPES (pH 7.35), 1 mM sodium pyruvate, 1 mM each of nonessential amino acids, 100 units/ml penicillin, 100 μg/ml streptomycin, 2 mM L-Glutamine and 55 μM 2-Mercaptoethanol (all supplements were from Invitrogen) at 37°C in 5% CO_2_. Cells were transiently transfected with the indicated plasmids using the FuGene6 Transfection Reagent (Roche, Indianapolis, IN) following the manufacturer's protocol.

### Antibodies and other reagents

The following antibodies were obtained from commercial sources: rabbit polyclonal (pAb) anti-EGFR (1005), pAb anti-phospho-AKT (pAKT1/2/3) (Ser 473), pAb anti-phospho-Erk 1/2 (Thr 202/Tyr 204), pAb anti-Erk1 (K-23), and pAb anti-Src (SRC 2) were from Santa Cruz Biotechnology (Santa Cruz, CA); mouse monoclonal (mAb) anti-phospho-EGFR (activated form) was from BD Biosciences (San Jose, CA); pAb anti-phospho-Src (Tyr416), pAb anti-phospho-EGFR (Tyr1173), pAb anti-STAT3, Rabbit monoclonal anti-phospho-STAT3 (Tyr705), and pAb anti-phospho-EGFR (Tyr845) were from Cell Signaling Technology (Danvers, MA); mAb anti-β actin (Clone AC-15) was from Sigma-Aldrich (St Louis, MO); mAb anti-LAMP1 (H4A3) was from Developmental Studies Hybridoma Bank (Iowa City, IA); mAb anti-EGFR (clone 528; ATCC) was Protein G purified from hybridoma supernatants. Purified anti-phosphotyrosine mAb 4G10 [71] was provided by Dr. Brian Druker (Oregon Health Science University, Portland, OR). Purified mouse EGF, human holo-Transferrin, and monensin were from Sigma-Aldrich. EGFR inhibitor, Erlotinib (Tarceva), was obtained from the Hospital Pharmacy. Src inhibitor PP2 was from Calbiochem (San Diego, CA). Hsp90 inhibitor 17-AAG was from Biomol International (Plymouth, PA, U.S.A.).

### Preparation of cell lysates, SDS-PAGE and immunoblotting

Cells at 50-60% confluence were incubated in normal growth medium, growth factor-deprived D3 medium (HBE135) [72] or 0.1% FBS-containing medium (H1666, H1650, HCC827, HCC4006 and H1975) for 48 hr. For EGF stimulation, cells preincubated in growth factor-deficient medium were either left as such or EGF was added at 10 ng/ml 10 min before cell lysis. Cell lysates were prepared in cold Triton X-100-based lysis buffer [7], and SDS-PAGE and immunoblotting were performed as previously described [7].

### Immunoprecipitation

Cells were grown, EGF stimulation performed, and cell lysates prepared as above with the exception that the lysis buffer contained 0.25% NP-40 (instead of 0.5% Triton X-100), 50 mM Tris (pH 8.0), and 100 mM sodium chloride. Cell lysate aliquots were incubated with anti-EGFR (528) antibody, and immune complexes were captured using Protein A-Sepharose beads (GE Healthcare, Piscataway, NJ). Subsequent SDS-PAGE and immunoblotting were performed as described above.

### Immunofluorescence microscopy

Cells were plated on glass coverslips (VWR, Batavia, IL) at 50-60% confluence and incubated in normal growth medium or growth factor-deficient medium for 48 hr. Cells were either left unstimulated or stimulated with EGF (10 ng/ml) for 30 min, washed in phosphate buffered saline (PBS, Cellgro, Manassas, VA), fixed in 3.7% formaldehyde (Sigma) in PBS for 20 min at RT, blocked in 2% FBS/PBS/0.02% sodium azide at 4°C for 24 hr, and permeabilized in immunostaining buffer with 0.05% Saponin (Sigma) and 0.2% BSA (Sigma) in PBS for 15 min. Cells were stained with primary antibodies diluted in immunostaining buffer for 1 hr and with Alexa 488- or Alexa 647-conjugated goat anti-mouse or goat anti-rabbit secondary antibodies (Invitrogen) for 1 hr. Coverslips were mounted on microscope slides with VECTASHIELD^® ^Hard Set™ Mounting Medium with DAPI (Vector Laboratories, Burlingame, CA). Confocal fluorescence images were obtained with a LSM510 fluorescence confocal microscope (Carl Zeiss, Thornwood, NY) under a 63× oil immersion lens. Colocalization coefficients for each channel were calculated using the LSM510 Image Examiner software. Colocalization parameters were either set automatically by the software or thresholds were set using the scattergrams. Colocalization coefficients from at least three images were obtained, and averages were either represented as percentages or normalized and plotted with standard deviation as error bars.

### Monensin Treatment

For analyses involving immunoblotting or immunoprecipitation, cells were starved in D3 or 0.1% FBS-containing media and preincubated in DMSO (0.1%) or 10 μM monensin for 4 hr. Cells were then continued as such or EGF (10 ng/ml) was added for 30 min followed by cell lysis. For immunofluorescence analyses, starved cells were preincubated in DMSO or monensin as above and loaded with 10 ug/ml of Alexa Fluor 546-conjugated transferrin (Invitrogen) for 45 min. Cells were then washed twice in PBS and either left unstimulated or stimulated with EGF (10 ng/ml) for 30 min. Cells were immunostained as described above.

## Abbreviations

EGF: Epidermal growth factor; EGFR: Epidermal growth factor receptor; LAMP1: Lysosomal-associated membrane protein 1; NSCLC: Non small cell lung cancer; RTK: Receptor tyrosine kinase; Tf: transferrin; TKI: Tyrosine kinase inhibitor; WT: Wild-type.

## Competing interests

The authors declare that they have no competing interests.

## Authors' contributions

HB and VB conceived the study and established the initial design of studies. BMC, and SMR carried out the experimental work including design alterations and data analysis in consultation with HB. RJC, CT and MG participated in data analysis and provided critical comments on the study design and manuscript. BMC prepared the draft of the manuscript and HB edited and finalized it. All authors read and approved the final manuscript.

## Note

^@^The work presented here was initiated while the investigators were in the Department of Medicine, Evanston Northwestern Healthcare (now Northshore University HealthSystem) Research Institute, Feinberg School of Medicine, Northwestern University, Evanston, IL, USA.

## Supplementary Material

Additional file 1 **Constitutive intracellular vesicular localization of mutant EGF receptors stably expressed in HBEC cell line**. (A) Cells were growth factor-deprived by culturing in D3 medium for 48 hr and then treated with 100 ng/ml EGF for indicated time periods. Cells were fixed, and GFP images were acquired under a confocal microscope at the medial plane. Bar represents 20 μm. (B) Cells were growth factor-deprived as in (A) for 48 hr and either left unstimulated (-) or stimulated (+) with 100 ng/ml EGF for 10 min. The indicated amounts of whole cell lysate protein were used for immunoblotting with antibodies against the indicated proteins. Exogenous EGFR-GFPs (EGFR-GFP) and endogenous EGFR (endogenous EGFR) are indicated with arrows. Click here for file

Additional file 2 **Enhanced colocalization of mutant EGF receptors with labeled transferrin upon incubation of cells at 16°C**. Cells were growth factor-deprived in 0.1% FBS-containing growth medium for 48 hr and preincubated at 37 or 16°C for 2 hr. Cells were loaded with 10 μg/ml AF546-Tf (Tf) (red) for 45 min. This was followed by incubation in growth factor-deprived medium with (+ EGF) or without (- EGF) 10 ng/ml EGF for 30 min. Cells were fixed, permeabilized, and immunostained with anti-EGFR antibody 528 (green). Images were acquired under a confocal microscope at the medial plane. Bars represent 10 μm. Click here for file

Additional file 3 **Monensin treatment inhibits the exit of labeled transferrin and mutant EGFR from the perinuclear endocytic recycling compartment**. (A) H1666 cells were growth factor deprived for 48 hr and preincubated with DMSO or 10 μM monensin for 3 hr. Cells were loaded with 10 μg/ml AF546-Tf (Tf) (red) for 30 min at 37°C and then chased in the presence of 2 mg/ml holo-transferrin for 0, 30 or 60 min in the continued presence of DMSO or 10 μM monensin. Cells were fixed, permeabilized and immunostained with anti-LAMP1 antibody (green). Images were acquired under a confocal microscope at the medial plane. (B) Cells were grown in regular growth medium and preincubated with DMSO or 10 μM monensin for 3 hr. Cells were loaded with 10 ug/ml AF546-Tf (Tf) (red) for 45 min at 37°C (with continuation of DMSO or monensin treatment). This was followed by incubation with regular growth medium for 30 min with continued DMSO or monensin treatment. Cells were fixed, permeabilized, and immunostained with anti-EGFR antibody 528 (green). Images were acquired under a confocal microscope at the medial plane. (C) HBEC cells stably expressing mutant EGF receptors were growth factor-deprived in D3 medium for 48 hr and then preincubated with DMSO or 10 μM monensin for 3 hr. Cells were incubated with (+EGF) or without (-EGF) 10 ng/ml EGF for 30 min with continued DMSO or monensin treatment. Cells were fixed, and GFP images were acquired under a confocal microscope at the medial plane. Bars represent 20 μm. Click here for file

Additional file 4 **Monensin treatment prevents lysosomal targeting and degradation of mutant EGFR by 17-AAG**. H1650 cells were grown in regular growth medium and preincubated with DMSO or 10 μM monensin for 4 hr. Cells were either left untreated or treated with 1 μM 17-AAG for 3 hr. Cells were fixed, permeabilized, and immunostained with anti-EGFR antibody 528 (green) followed by anti-LAMP1 antibody (red). Images were acquired under a confocal microscope at the medial plane. Bars represent 20 μm. Click here for file

Additional file 5 **Monensin treatment does not alter the overall levels of phosphorylation or expression of EGFR and its downstream factors**. Cells were growth factor-deprived in D3 medium (HBE135) or 0.1% FBS-containing growth medium (all other cell lines) for 48 hr and preincubated with DMSO or 10 μM monensin for 3 hr. Cells were then either left unstimulated (-) or stimulated (+) with 10 ng/ml EGF for 30 min. 50 μg aliquots of WCL were used for immunoblotting with antibodies against the indicated proteins. Click here for file

Additional file 6 **Src inhibitor does not alter intracellular localization of mutant EGFR but reduces mutant EGFR-Src association**. (A) HCC827 cells were growth factor deprived for 48 hr and preincubated with DMSO, 1 μM Erlotinib (ER) or 3 μM PP2 for 3 hr. Cells were fixed, permeabilized, and immunostained with anti-EGFR antibody 528 (green). Images were acquired under a confocal microscope at the medial plane. Bars represent 20 μm. (B) HCC827 cells were grown in regular growth medium and preincubated with DMSO, 1 μM Erlotinib (ER) or 3 μM PP2 for 3 hr. 1 mg aliquots of cell lysate proteins were immunoprecipitated with anti-EGFR antibody 528 (EGFR IP) and resolved together with 50 μg aliquots of whole cell lysates (WCL) followed by immunoblotting with antibodies against the indicated proteins. Click here for file

## References

[B1] Herbst RS Review of epidermal growth factor receptor biology Int J Radiat Oncol Biol Phys 2004 59 2 Suppl 21 26 15142631 10.1016/j.ijrobp.2003.11.041

[B2] Yarden Y Sliwkowski MX Untangling the ErbB signalling network Nat Rev Mol Cell Biol 2001 2 2 127 137 10.1038/35052073 11252954

[B3] Sharma SV Bell DW Settleman J Haber DA Epidermal growth factor receptor mutations in lung cancer Nat Rev Cancer 2007 7 3 169 181 10.1038/nrc2088 17318210

[B4] Sordella R Bell DW Haber DA Settleman J Gefitinib-sensitizing EGFR mutations in lung cancer activate anti-apoptotic pathways Science 2004 305 5687 1163 1167 10.1126/science.1101637 15284455

[B5] Lynch TJ Bell DW Sordella R Gurubhagavatula S Okimoto RA Brannigan BW Harris PL Haserlat SM Supko JG Haluska FG Louis DN Christiani DC Settleman J Haber DA Activating mutations in the epidermal growth factor receptor underlying responsiveness of non-small-cell lung cancer to gefitinib N Engl J Med 2004 350 21 2129 2139 10.1056/NEJMoa040938 15118073

[B6] Paez JG Janne PA Lee JC Tracy S Greulich H Gabriel S Herman P Kaye FJ Lindeman N Boggon TJ Naoki K Sasaki H Fujii Y Eck MJ Sellers WR Johnson BE Meyerson M EGFR mutations in lung cancer: correlation with clinical response to gefitinib therapy Science 2004 304 5676 1497 1500 10.1126/science.1099314 15118125

[B7] Chung BM Dimri M George M Reddi AL Chen G Band V Band H The role of cooperativity with Src in oncogenic transformation mediated by non-small cell lung cancer-associated EGF receptor mutants Oncogene 2009 28 16 1821 1832 10.1038/onc.2009.31 19305428 PMC2752420

[B8] Engelman JA Janne PA Mermel C Pearlberg J Mukohara T Fleet C Cichowski K Johnson BE Cantley LC ErbB-3 mediates phosphoinositide 3-kinase activity in gefitinib-sensitive non-small cell lung cancer cell lines Proc Natl Acad Sci USA 2005 102 10 3788 3793 10.1073/pnas.0409773102 15731348 PMC553328

[B9] Zhang J Kalyankrishna S Wislez M Thilaganathan N Saigal B Wei W Ma L Wistuba II Johnson FM Kurie JM SRC-family kinases are activated in non-small cell lung cancer and promote the survival of epidermal growth factor receptor-dependent cell lines Am J Pathol 2007 170 1 366 376 10.2353/ajpath.2007.060706 17200208 PMC1762707

[B10] Song L Morris M Bagui T Lee FY Jove R Haura EB Dasatinib (BMS-354825) selectively induces apoptosis in lung cancer cells dependent on epidermal growth factor receptor signaling for survival Cancer Res 2006 66 11 5542 5548 10.1158/0008-5472.CAN-05-4620 16740687

[B11] Fu YN Yeh CL Cheng HH Yang CH Tsai SF Huang SF Chen YR EGFR mutants found in non-small cell lung cancer show different levels of sensitivity to suppression of Src: implications in targeting therapy Oncogene 2008 27 7 957 965 10.1038/sj.onc.1210684 17653080

[B12] Mosesson Y Mills GB Yarden Y Derailed endocytosis: an emerging feature of cancer Nat Rev Cancer 2008 8 11 835 850 10.1038/nrc2521 18948996

[B13] Maxfield FR McGraw TE Endocytic recycling Nat Rev Mol Cell Biol 2004 5 2 121 132 10.1038/nrm1315 15040445

[B14] Levkowitz G Waterman H Zamir E Kam Z Oved S Langdon WY Beguinot L Geiger B Yarden Y c-Cbl/Sli-1 regulates endocytic sorting and ubiquitination of the epidermal growth factor receptor Genes Dev 1998 12 23 3663 3674 10.1101/gad.12.23.3663 9851973 PMC317257

[B15] Duan L Miura Y Dimri M Majumder B Dodge IL Reddi AL Ghosh A Fernandes N Zhou P Mullane-Robinson K Rao N Donoghue S Rogers RA Bowtell D Naramura M Gu H Band V Band H Cbl-mediated ubiquitinylation is required for lysosomal sorting of epidermal growth factor receptor but is dispensable for endocytosis J Biol Chem 2003 278 31 28950 28960 10.1074/jbc.M304474200 12754251

[B16] Katzmann DJ Odorizzi G Emr SD Receptor downregulation and multivesicular-body sorting Nat Rev Mol Cell Biol 2002 3 12 893 905 10.1038/nrm973 12461556

[B17] Pennock S Wang Z A tale of two Cbls: interplay of c-Cbl and Cbl-b in epidermal growth factor receptor downregulation Mol Cell Biol 2008 28 9 3020 3037 10.1128/MCB.01809-07 18316398 PMC2293090

[B18] Kowanetz K Crosetto N Haglund K Schmidt MH Heldin CH Dikic I Suppressors of T-cell receptor signaling Sts-1 and Sts-2 bind to Cbl and inhibit endocytosis of receptor tyrosine kinases J Biol Chem 2004 279 31 32786 32795 10.1074/jbc.M403759200 15159412

[B19] Lynch DK Winata SC Lyons RJ Hughes WE Lehrbach GM Wasinger V Corthals G Cordwell S Daly RJ A Cortactin-CD2-associated protein (CD2AP) complex provides a novel link between epidermal growth factor receptor endocytosis and the actin cytoskeleton J Biol Chem 2003 278 24 21805 21813 10.1074/jbc.M211407200 12672817

[B20] Stang E Blystad FD Kazazic M Bertelsen V Brodahl T Raiborg C Stenmark H Madshus IH Cbl-dependent ubiquitination is required for progression of EGF receptors into clathrin-coated pits Mol Biol Cell 2004 15 8 3591 3604 10.1091/mbc.E04-01-0041 15194809 PMC491821

[B21] Worby CA Dixon JE Sorting out the cellular functions of sorting nexins Nat Rev Mol Cell Biol 2002 3 12 919 931 10.1038/nrm974 12461558

[B22] Alwan HA van Zoelen EJ van Leeuwen JE Ligand-induced lysosomal epidermal growth factor receptor (EGFR) degradation is preceded by proteasome-dependent EGFR de-ubiquitination J Biol Chem 2003 278 37 35781 35790 10.1074/jbc.M301326200 12829707

[B23] Stern KA Visser Smit GD Place TL Winistorfer S Piper RC Lill NL Epidermal growth factor receptor fate is controlled by Hrs tyrosine phosphorylation sites that regulate Hrs degradation Mol Cell Biol 2007 27 3 888 898 10.1128/MCB.02356-05 17101784 PMC1800687

[B24] Conner SD Schmid SL Regulated portals of entry into the cell Nature 2003 422 6927 37 44 10.1038/nature01451 12621426

[B25] Hendriks BS Wiley HS Lauffenburger D HER2-mediated effects on EGFR endosomal sorting: analysis of biophysical mechanisms Biophys J 2003 85 4 2732 2745 10.1016/S0006-3495(03)74696-7 14507736 PMC1303497

[B26] Shankaran H Zhang Y Opresko L Resat H Quantifying the effects of co-expressing EGFR and HER2 on HER activation and trafficking Biochem Biophys Res Commun 2008 371 2 220 224 10.1016/j.bbrc.2008.04.043 18424261 PMC2864016

[B27] Offterdinger M Bastiaens PI Prolonged EGFR signaling by ERBB2-mediated sequestration at the plasma membrane Traffic 2008 9 1 147 155 10.1111/j.1600-0854.2007.00665.x 17956594

[B28] Shtiegman K Kochupurakkal BS Zwang Y Pines G Starr A Vexler A Citri A Katz M Lavi S Ben-Basat Y Benjamin S Corso S Gan J Yosef RB Giordano S Yarden Y Defective ubiquitinylation of EGFR mutants of lung cancer confers prolonged signaling Oncogene 2007 26 49 6968 6978 10.1038/sj.onc.1210503 17486068

[B29] Padron D Sato M Shay JW Gazdar AF Minna JD Roth MG Epidermal growth factor receptors with tyrosine kinase domain mutations exhibit reduced Cbl association, poor ubiquitylation, and down-regulation but are efficiently internalized Cancer Res 2007 67 16 7695 7702 10.1158/0008-5472.CAN-07-0484 17699773 PMC2852256

[B30] Sigismund S Argenzio E Tosoni D Cavallaro E Polo S Di Fiore PP Clathrin-mediated internalization is essential for sustained EGFR signaling but dispensable for degradation Dev Cell 2008 15 2 209 219 10.1016/j.devcel.2008.06.012 18694561

[B31] Choi SH Mendrola JM Lemmon MA EGF-independent activation of cell-surface EGF receptors harboring mutations found in gefitinib-sensitive lung cancer Oncogene 2007 26 11 1567 1576 10.1038/sj.onc.1209957 16953218

[B32] Nishimura Y Bereczky B Ono M The EGFR inhibitor gefitinib suppresses ligand-stimulated endocytosis of EGFR via the early/late endocytic pathway in non-small cell lung cancer cell lines Histochem Cell Biol 2007 127 5 541 553 10.1007/s00418-007-0281-y 17361439

[B33] Engelman JA Zejnullahu K Gale CM Lifshits E Gonzales AJ Shimamura T Zhao F Vincent PW Naumov GN Bradner JE Althaus IW Gandhi L Shapiro GI Nelson JM Heymach JV Meyerson M Wong KK Janne PA PF0029 an irreversible pan-ERBB inhibitor, is effective in lung cancer models with EGFR and ERBB2 mutations that are resistant to gefitinib Cancer Res 9804 67 24 11924 11932 10.1158/0008-5472.CAN-07-1885 18089823

[B34] Wang Y Pennock S Chen X Wang Z Endosomal signaling of epidermal growth factor receptor stimulates signal transduction pathways leading to cell survival Mol Cell Biol 2002 22 20 7279 7290 10.1128/MCB.22.20.7279-7290.2002 12242303 PMC139821

[B35] Zhang X Gureasko J Shen K Cole PA Kuriyan J An allosteric mechanism for activation of the kinase domain of epidermal growth factor receptor Cell 2006 125 6 1137 1149 10.1016/j.cell.2006.05.013 16777603

[B36] Ren M Xu G Zeng J De Lemos-Chiarandini C Adesnik M Sabatini DD Hydrolysis of GTP on rab11 is required for the direct delivery of transferrin from the pericentriolar recycling compartment to the cell surface but not from sorting endosomes Proc Natl Acad Sci USA 1998 95 11 6187 6192 10.1073/pnas.95.11.6187 9600939 PMC27621

[B37] Yang S Qu S Perez-Tores M Sawai A Rosen N Solit DB Arteaga CL Association with HSP90 inhibits Cbl-mediated down-regulation of mutant epidermal growth factor receptors Cancer Res 2006 66 14 6990 6997 10.1158/0008-5472.CAN-06-1042 16849543

[B38] Stein BS Bensch KG Sussman HH Complete inhibition of transferrin recycling by monensin in K562 cells J Biol Chem 1984 259 23 14762 14772 6094573

[B39] Tran DD Russell HR Sutor SL van Deursen J Bram RJ CAML is required for efficient EGF receptor recycling Dev Cell 2003 5 2 245 256 10.1016/S1534-5807(03)00207-7 12919676

[B40] Raja SM Clubb RJ Bhattacharyya M Dimri M Cheng H Pan W Ortega-Cava C Lakku-Reddi A Naramura M Band V Band H A combination of Trastuzumab and 17-AAG induces enhanced ubiquitinylation and lysosomal pathway-dependent ErbB2 degradation and cytotoxicity in ErbB2-overexpressing breast cancer cells Cancer Biol Ther 2008 7 10 1630 1640 18769124 10.4161/cbt.7.10.6585 PMC2727620

[B41] Shimamura T Lowell AM Engelman JA Shapiro GI Epidermal growth factor receptors harboring kinase domain mutations associate with the heat shock protein 90 chaperone and are destabilized following exposure to geldanamycins Cancer Res 2005 65 14 6401 6408 10.1158/0008-5472.CAN-05-0933 16024644

[B42] Wileman T Boshans RL Schlesinger P Stahl P Monensin inhibits recycling of macrophage mannose-glycoprotein receptors and ligand delivery to lysosomes Biochem J 1984 220 3 665 675 6087792 10.1042/bj2200665 PMC1153682

[B43] George M Ying G Rainey MA Solomon A Parikh PT Gao Q Band V Band H Shared as well as distinct roles of EHD proteins revealed by biochemical and functional comparisons in mammalian cells and C. elegans BMC Cell Biol 2007 8 3 10.1186/1471-2121-8-3 17233914 PMC1793994

[B44] Grant BD Caplan S Mechanisms of EHD/RME-1 protein function in endocytic transport Traffic 2008 9 12 2043 2052 10.1111/j.1600-0854.2008.00834.x 18801062 PMC2766864

[B45] Donepudi M Resh MD c-Src trafficking and co-localization with the EGF receptor promotes EGF ligand-independent EGF receptor activation and signaling Cell Signal 2008 20 7 1359 1367 10.1016/j.cellsig.2008.03.007 18448311 PMC2459337

[B46] Kaplan KB Swedlow JR Varmus HE Morgan DO Association of p60c-src with endosomal membranes in mammalian fibroblasts J Cell Biol 1992 118 2 321 333 10.1083/jcb.118.2.321 1378446 PMC2290043

[B47] Bjorge JD Jakymiw A Fujita DJ Selected glimpses into the activation and function of Src kinase Oncogene 2000 19 49 5620 5635 10.1038/sj.onc.1203923 11114743

[B48] Yang S Park K Turkson J Arteaga CL Ligand-independent phosphorylation of Y869(Y845) links mutant EGFR signaling to stat-mediated gene expression Exp Cell Res 2008 314 2 413 419 10.1016/j.yexcr.2007.09.002 17927978 PMC3221731

[B49] Citri A Yarden Y EGF-ERBB signalling: towards the systems level Nat Rev Mol Cell Biol 2006 7 7 505 516 10.1038/nrm1962 16829981

[B50] Wang Q Villeneuve G Wang Z Control of epidermal growth factor receptor endocytosis by receptor dimerization, rather than receptor kinase activation EMBO Rep 2005 6 10 942 948 10.1038/sj.embor.7400491 16113650 PMC1369181

[B51] Wang Z Moran MF Requirement for the adapter protein GRB2 in EGF receptor endocytosis Science 1996 272 5270 1935 1939 10.1126/science.272.5270.1935 8658166

[B52] Marmor MD Yarden Y Role of protein ubiquitylation in regulating endocytosis of receptor tyrosine kinases Oncogene 2004 23 11 2057 2070 10.1038/sj.onc.1207390 15021893

[B53] Furukawa M Nagatomo I Kumagai T Yamadori T Takahashi R Yoshimura M Yoneda T Takeda Y Goya S Matsuoka H Kijima T Yoshida M Osaki T Tachibana I Greene MI Kawase I Gefitinib-sensitive EGFR lacking residues 746-750 exhibits hypophosphorylation at tyrosine residue 1045, hypoubiquitination, and impaired endocytosis DNA Cell Biol 2007 26 3 178 185 10.1089/dna.2006.0573 17417946

[B54] Nishimura Y Yoshioka K Bereczky B Itoh K Evidence for efficient phosphorylation of EGFR and rapid endocytosis of phosphorylated EGFR via the early/late endocytic pathway in a gefitinib-sensitive non-small cell lung cancer cell line Mol Cancer 2008 7 42 10.1186/1476-4598-7-42 18492291 PMC2412912

[B55] Grovdal LM Stang E Sorkin A Madshus IH Direct interaction of Cbl with pTyr 1045 of the EGF receptor (EGFR) is required to sort the EGFR to lysosomes for degradation Exp Cell Res 2004 300 2 388 395 10.1016/j.yexcr.2004.07.003 15475003

[B56] Ravid T Heidinger JM Gee P Khan EM Goldkorn T c-Cbl-mediated ubiquitinylation is required for epidermal growth factor receptor exit from the early endosomes J Biol Chem 2004 279 35 37153 37162 10.1074/jbc.M403210200 15210722

[B57] Longva KE Blystad FD Stang E Larsen AM Johannessen LE Madshus IH Ubiquitination and proteasomal activity is required for transport of the EGF receptor to inner membranes of multivesicular bodies J Cell Biol 2002 156 5 843 854 10.1083/jcb.200106056 11864992 PMC2173306

[B58] Mizuno E Iura T Mukai A Yoshimori T Kitamura N Komada M Regulation of epidermal growth factor receptor down-regulation by UBPY-mediated deubiquitination at endosomes Mol Biol Cell 2005 16 11 5163 5174 10.1091/mbc.E05-06-0560 16120644 PMC1266416

[B59] Niendorf S Oksche A Kisser A Lohler J Prinz M Schorle H Feller S Lewitzky M Horak I Knobeloch KP Essential role of ubiquitin-specific protease 8 for receptor tyrosine kinase stability and endocytic trafficking in vivo Mol Cell Biol 2007 27 13 5029 5039 10.1128/MCB.01566-06 17452457 PMC1951504

[B60] Aoh QL Castle AM Hubbard CH Katsumata O Castle JD SCAMP3 Negatively Regulates EGFR Degradation and Promotes Receptor Recycling Mol Biol Cell 2009 20 6 1816 1832 10.1091/mbc.E08-09-0894 19158374 PMC2655259

[B61] Sakai K Arao T Shimoyama T Murofushi K Sekijima M Kaji N Tamura T Saijo N Nishio K Dimerization and the signal transduction pathway of a small in-frame deletion in the epidermal growth factor receptor FASEB J 2006 20 2 311 313 16373402 10.1096/fj.05-4034fje

[B62] Tao RH Maruyama IN All EGF(ErbB) receptors have preformed homo- and heterodimeric structures in living cells J Cell Sci 2008 121 Pt 19 3207 3217 10.1242/jcs.033399 18782861

[B63] Vieira AV Lamaze C Schmid SL Control of EGF receptor signaling by clathrin-mediated endocytosis Science 1996 274 5295 2086 2089 10.1126/science.274.5295.2086 8953040

[B64] Pennock S Wang Z Stimulation of cell proliferation by endosomal epidermal growth factor receptor as revealed through two distinct phases of signaling Mol Cell Biol 2003 23 16 5803 5815 10.1128/MCB.23.16.5803-5815.2003 12897150 PMC166318

[B65] Yin HS Yang MF Effect of monensin on the neuronal ultrastructure and endocytic pathway of macromolecules in cultured brain neurons Cell Mol Neurobiol 1992 12 4 297 307 10.1007/BF00734930 1394368 PMC11567485

[B66] Sandilands E Frame MC Endosomal trafficking of Src tyrosine kinase Trends Cell Biol 2008 18 7 322 329 10.1016/j.tcb.2008.05.004 18515107

[B67] Kwak EL Sordella R Bell DW Godin-Heymann N Okimoto RA Brannigan BW Harris PL Driscoll DR Fidias P Lynch TJ Rabindran SK McGinnis JP Wissner A Sharma SV Isselbacher KJ Settleman J Haber DA Irreversible inhibitors of the EGF receptor may circumvent acquired resistance to gefitinib Proc Natl Acad Sci USA 2005 102 21 7665 7670 10.1073/pnas.0502860102 15897464 PMC1129023

[B68] Guo A Villen J Kornhauser J Lee KA Stokes MP Rikova K Possemato A Nardone J Innocenti G Wetzel R Wang Y MacNeill J Mitchell J Gygi SP Rush J Polakiewicz RD Comb MJ Signaling networks assembled by oncogenic EGFR and c-Met Proc Natl Acad Sci USA 2008 105 2 692 697 10.1073/pnas.0707270105 18180459 PMC2206598

[B69] Powelka AM Sun J Li J Gao M Shaw LM Sonnenberg A Hsu VW Stimulation-dependent recycling of integrin beta1 regulated by ARF6 and Rab11 Traffic 2004 5 1 20 36 10.1111/j.1600-0854.2004.00150.x 14675422

[B70] Band V Sager R Distinctive traits of normal and tumor-derived human mammary epithelial cells expressed in a medium that supports long-term growth of both cell types Proc Natl Acad Sci USA 1989 86 4 1249 1253 10.1073/pnas.86.4.1249 2919173 PMC286665

[B71] Druker BJ Mamon HJ Roberts TM Oncogenes, growth factors, and signal transduction N Engl J Med 1989 321 20 1383 1391 2682241 10.1056/NEJM198911163212007

[B72] Dimri M Naramura M Duan L Chen J Ortega-Cava C Chen G Goswami R Fernandes N Gao Q Dimri GP Band V Band H Modeling breast cancer-associated c-Src and EGFR overexpression in human MECs: c-Src and EGFR cooperatively promote aberrant three-dimensional acinar structure and invasive behavior Cancer Res 2007 67 9 4164 4172 10.1158/0008-5472.CAN-06-2580 17483327

