# Supplementary figures and images for: Aberrant trafficking of NSCLC-associated EGFR mutants through the endocytic recycling pathway promotes interaction with Src@
Source: BMC Cell Biol. 2009 Nov 30;10:84. doi: 10.1186/1471-2121-10-84 (PMC2790444; doi:10.1186/1471-2121-10-84)

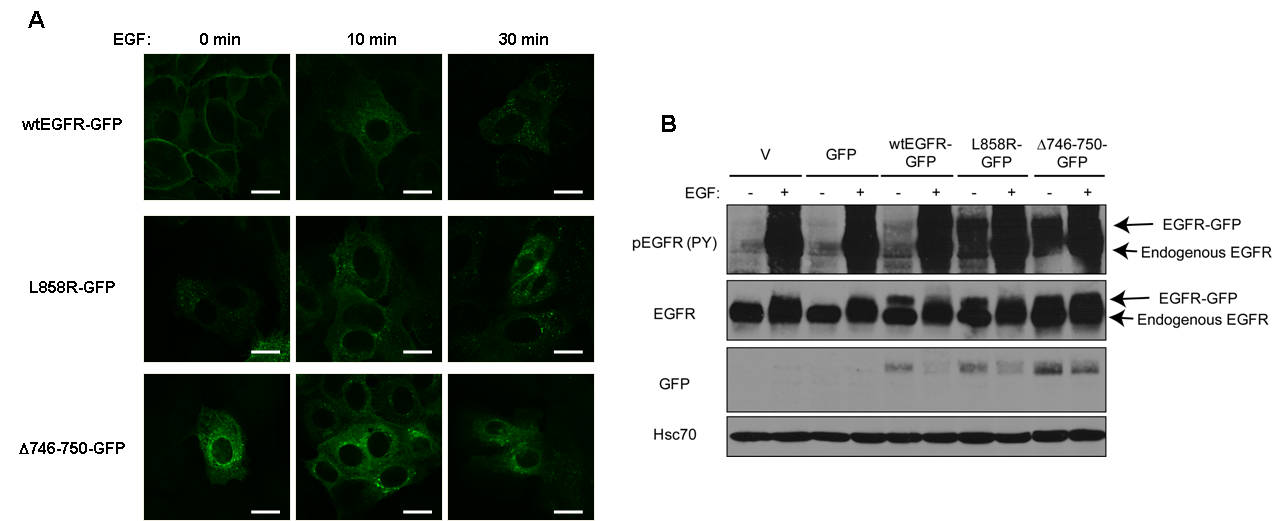

Supplement: Additional file 1 — Constitutive intracellular vesicular localization of mutant EGF receptors stably expressed in HBEC cell line. (A) Cells were growth factor-deprived by culturing in D3 medium for 48 hr and then treated with 100 ng/ml EGF for indicated time periods. Cells were fixed, and GFP images were acquired under a confocal microscope at the medial plane. Bar represents 20 μm. (B) Cells were growth factor-deprived as in (A) for 48 hr and either left unstimulated (-) or stimulated (+) with 100 ng/ml EGF for 10 min. The indicated amounts of whole cell lysate protein were used for immunoblotting with antibodies against the indicated proteins. Exogenous EGFR-GFPs (EGFR-GFP) and endogenous EGFR (endogenous EGFR) are indicated with arrows. [file 1471-2121-10-84-S1.TIFF]

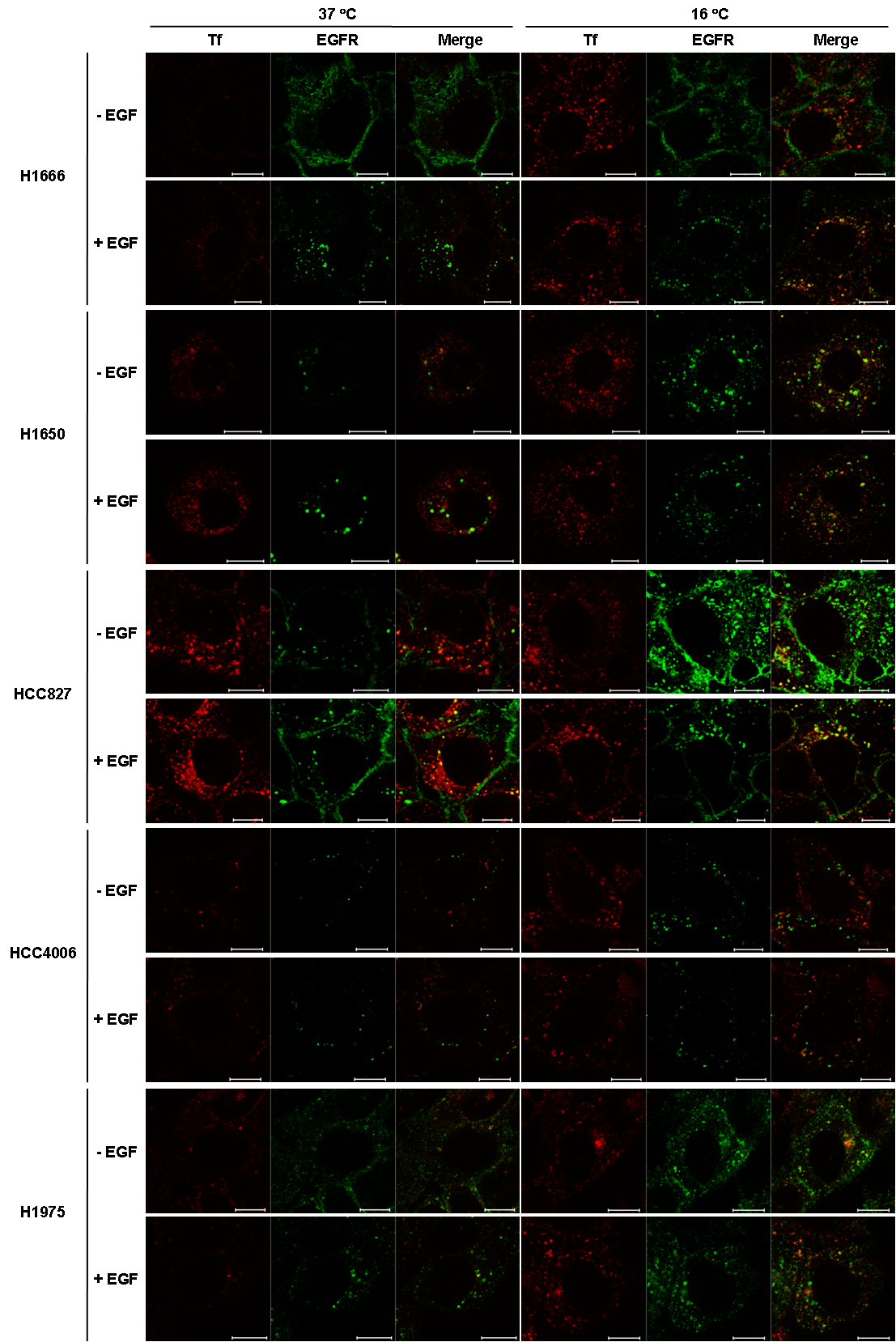

Supplement: Additional file 2 — Enhanced colocalization of mutant EGF receptors with labeled transferrin upon incubation of cells at 16°C. Cells were growth factor-deprived in 0.1% FBS-containing growth medium for 48 hr and preincubated at 37 or 16°C for 2 hr. Cells were loaded with 10 μg/ml AF546-Tf (Tf) (red) for 45 min. This was followed by incubation in growth factor-deprived medium with (+ EGF) or without (- EGF) 10 ng/ml EGF for 30 min. Cells were fixed, permeabilized, and immunostained with anti-EGFR antibody 528 (green). Images were acquired under a confocal microscope at the medial plane. Bars represent 10 μm. [file 1471-2121-10-84-S2.TIFF]

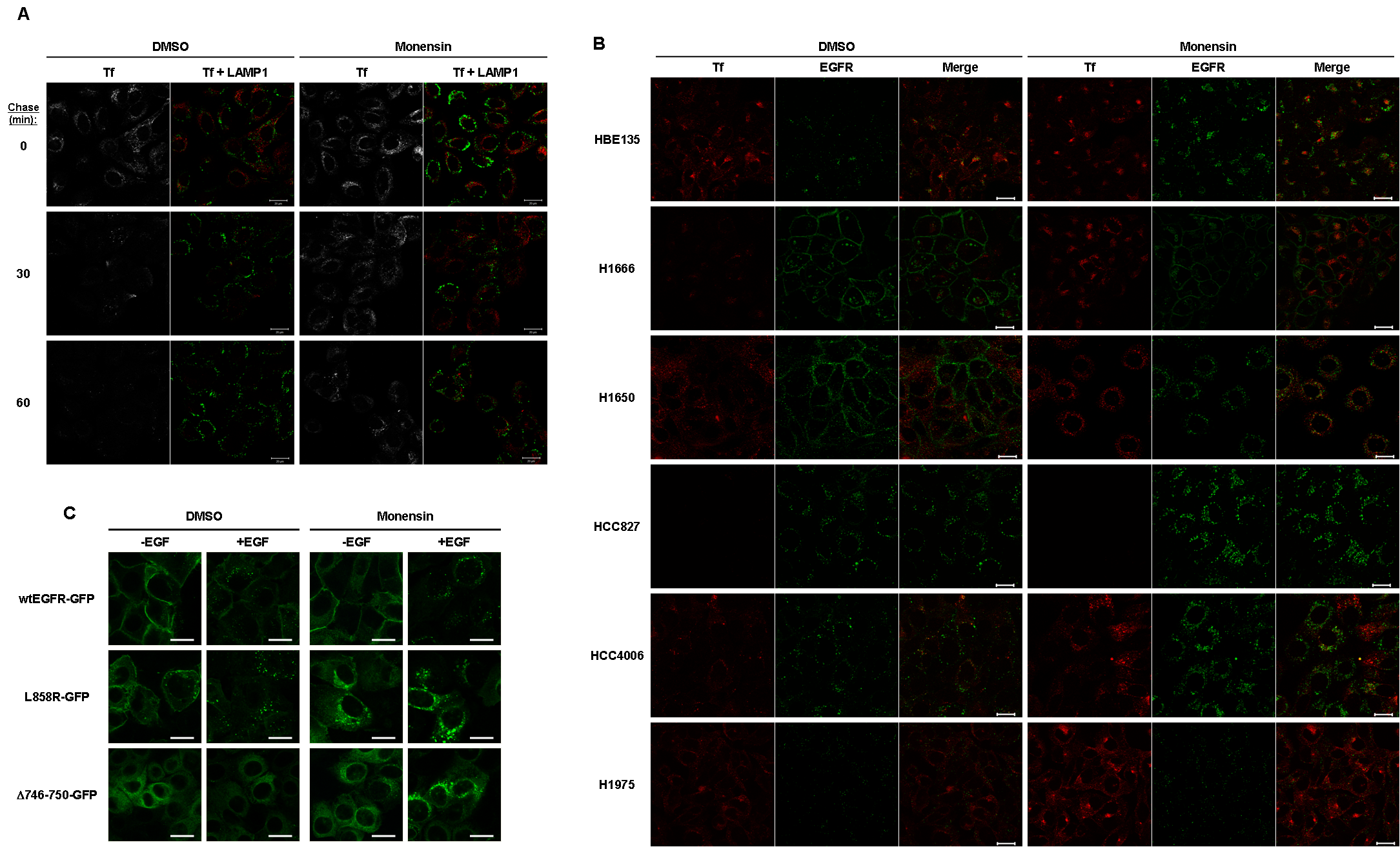

Supplement: Additional file 3 — Monensin treatment inhibits the exit of labeled transferrin and mutant EGFR from the perinuclear endocytic recycling compartment. (A) H1666 cells were growth factor deprived for 48 hr and preincubated with DMSO or 10 μM monensin for 3 hr. Cells were loaded with 10 μg/ml AF546-Tf (Tf) (red) for 30 min at 37°C and then chased in the presence of 2 mg/ml holo-transferrin for 0, 30 or 60 min in the continued presence of DMSO or 10 μM monensin. Cells were fixed, permeabilized and immunostained with anti-LAMP1 antibody (green). Images were acquired under a confocal microscope at the medial plane. (B) Cells were grown in regular growth medium and preincubated with DMSO or 10 μM monensin for 3 hr. Cells were loaded with 10 ug/ml AF546-Tf (Tf) (red) for 45 min at 37°C (with continuation of DMSO or monensin treatment). This was followed by incubation with regular growth medium for 30 min with continued DMSO or monensin treatment. Cells were fixed, permeabilized, and immunostained with anti-EGFR antibody 528 (green). Images were acquired under a confocal microscope at the medial plane. (C) HBEC cells stably expressing mutant EGF receptors were growth factor-deprived in D3 medium for 48 hr and then preincubated with DMSO or 10 μM monensin for 3 hr. Cells were incubated with (+EGF) or without (-EGF) 10 ng/ml EGF for 30 min with continued DMSO or monensin treatment. Cells were fixed, and GFP images were acquired under a confocal microscope at the medial plane. Bars represent 20 μm. [file 1471-2121-10-84-S3.TIFF]

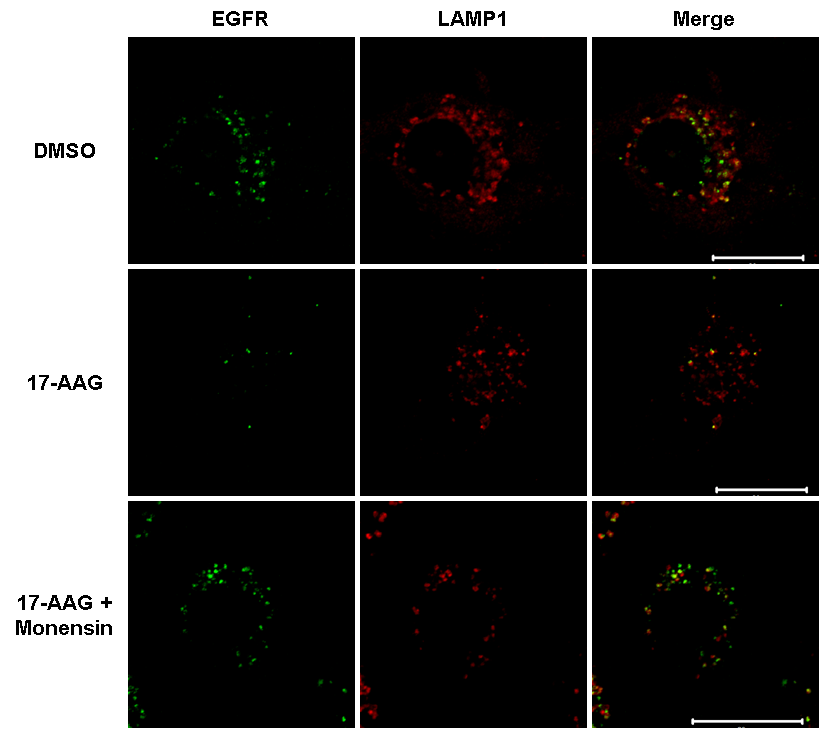

Supplement: Additional file 4 — Monensin treatment prevents lysosomal targeting and degradation of mutant EGFR by 17-AAG. H1650 cells were grown in regular growth medium and preincubated with DMSO or 10 μM monensin for 4 hr. Cells were either left untreated or treated with 1 μM 17-AAG for 3 hr. Cells were fixed, permeabilized, and immunostained with anti-EGFR antibody 528 (green) followed by anti-LAMP1 antibody (red). Images were acquired under a confocal microscope at the medial plane. Bars represent 20 μm. [file 1471-2121-10-84-S4.TIFF]

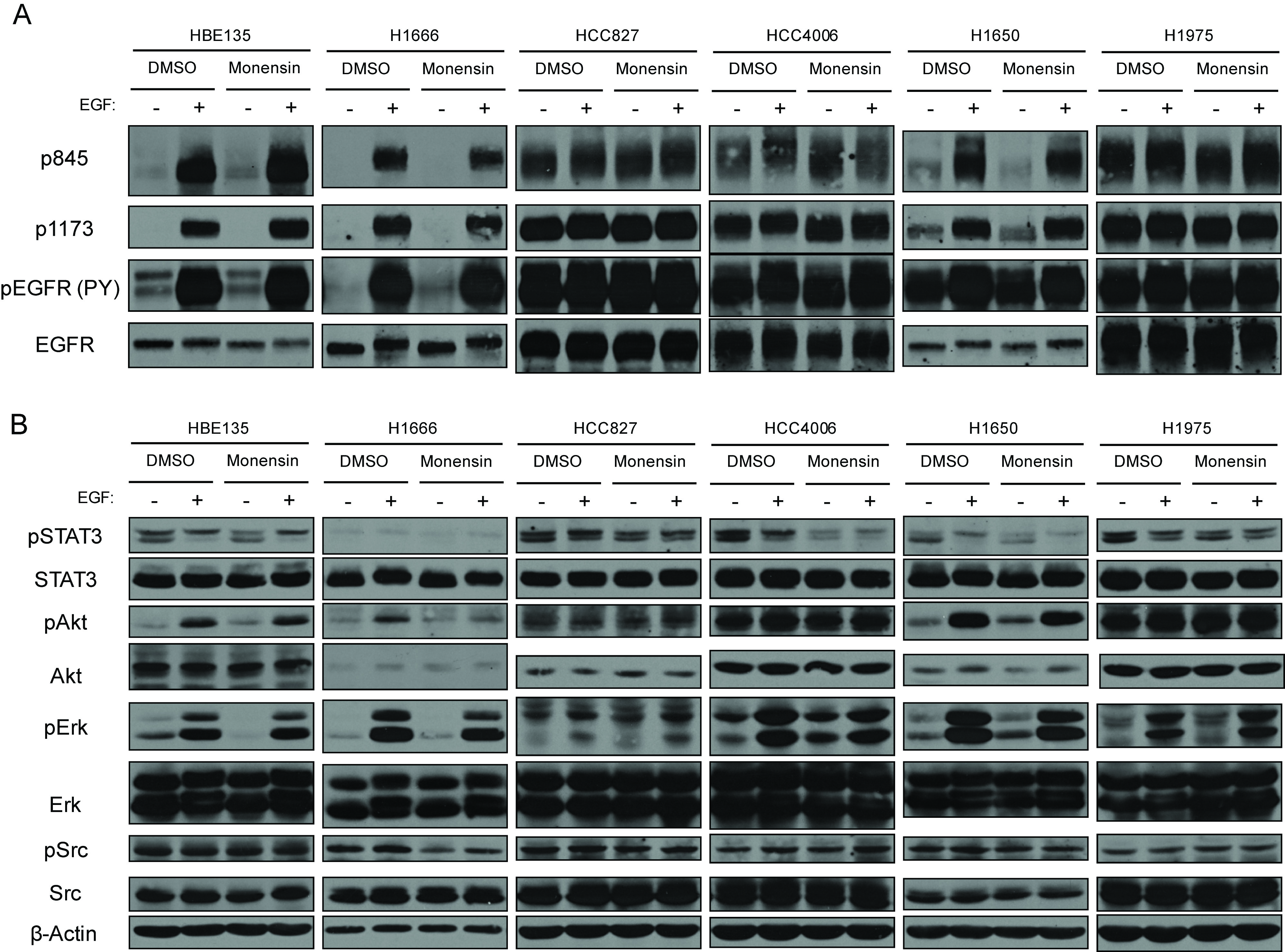

Supplement: Additional file 5 — Monensin treatment does not alter the overall levels of phosphorylation or expression of EGFR and its downstream factors. Cells were growth factor-deprived in D3 medium (HBE135) or 0.1% FBS-containing growth medium (all other cell lines) for 48 hr and preincubated with DMSO or 10 μM monensin for 3 hr. Cells were then either left unstimulated (-) or stimulated (+) with 10 ng/ml EGF for 30 min. 50 μg aliquots of WCL were used for immunoblotting with antibodies against the indicated proteins. [file 1471-2121-10-84-S5.JPEG]

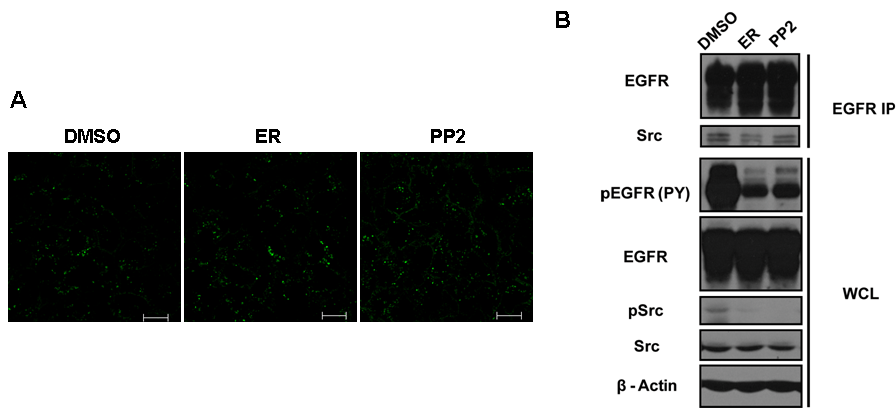

Supplement: Additional file 6 — Src inhibitor does not alter intracellular localization of mutant EGFR but reduces mutant EGFR-Src association. (A) HCC827 cells were growth factor deprived for 48 hr and preincubated with DMSO, 1 μM Erlotinib (ER) or 3 μM PP2 for 3 hr. Cells were fixed, permeabilized, and immunostained with anti-EGFR antibody 528 (green). Images were acquired under a confocal microscope at the medial plane. Bars represent 20 μm. (B) HCC827 cells were grown in regular growth medium and preincubated with DMSO, 1 μM Erlotinib (ER) or 3 μM PP2 for 3 hr. 1 mg aliquots of cell lysate proteins were immunoprecipitated with anti-EGFR antibody 528 (EGFR IP) and resolved together with 50 μg aliquots of whole cell lysates (WCL) followed by immunoblotting with antibodies against the indicated proteins. [file 1471-2121-10-84-S6.TIFF]
